# A Statewide Collaboration to Deliver and Evaluate a Pediatric Critical Care Simulation Curriculum for Emergency Medical Services

**DOI:** 10.3389/fped.2022.903950

**Published:** 2022-06-14

**Authors:** Caitlin Farrell, Kate Dorney, Bonnie Mathews, Tehnaz Boyle, Anthony Kitchen, Jeff Doyle, Michael C. Monuteaux, Joyce Li, Barbara Walsh, Joshua Nagler, Sarita Chung

**Affiliations:** ^1^Division of Emergency Medicine, Department of Pediatrics, Harvard Medical School, Boston, MA, United States; ^2^Division of Emergency Medicine, Department of Pediatrics, UMass Medical School, Worcester, MA, United States; ^3^Division of Emergency Medicine, Department of Pediatrics, Boston University School of Medicine, Boston, MA, United States; ^4^Department of Emergency Medicine, Baystate Medical Center, Springfield, MA, United States; ^5^Department of Public Health, Emergency Medical Services for Children, Boston, MA, United States

**Keywords:** simulation, pediatric critical care, prehospital resuscitation, emergency medical services, Emergency Medical Services for Children

## Abstract

**Objective:**

Care of the critically ill child is a rare but stressful event for emergency medical services (EMS) providers. Simulation training can improve resuscitation care and prehospital outcomes but limited access to experts, simulation equipment, and cost have limited adoption by EMS systems. Our objective was to form a statewide collaboration to develop, deliver, and evaluate a pediatric critical care simulation curriculum for EMS providers.

**Methods:**

We describe a statewide collaboration between five academic centers to develop a simulation curriculum and deliver it to EMS providers. Cases were developed by the collaborating PEM faculty, reviewed by EMS regional directors, and based on previously published EMS curricula, a statewide needs assessment, and updated state EMS protocols. The simulation curriculum was comprised of 3 scenarios requiring recognition and acute management of critically ill infants and children. The curriculum was implemented through 5 separate education sessions, led by a faculty lead at each site, over a 6 month time period. We evaluated curriculum effectiveness with a prospective, interventional, single-arm educational study using pre-post assessment design to assess the impact on EMS provider knowledge and confidence. To assess the intervention effect on knowledge scores while accounting for nested data, we estimated a mixed effects generalized regression model with random effects for region and participant. We assessed for knowledge retention and self-reported practice change at 6 months post-curriculum. Qualitative analysis of participants' written responses immediately following the curriculum and at 6 month follow-up was performed using the framework method.

**Results:**

Overall, 78 emergency medical technicians (EMTs) and 109 paramedics participated in the curriculum over five separate sessions. Most participants were male (69%) and paramedics (58%). One third had over 15 years of clinical experience. In the regression analysis, mean pediatric knowledge scores increased by 9.8% (95% CI: 7.2%, 12.4%). Most (93% [95% CI: 87.2%, 96.5%]) participants reported improved confidence caring for pediatric patients. Though follow-up responses were limited, participants who completed follow up surveys reported they had used skills acquired during the curriculum in clinical practice.

**Conclusion:**

Through statewide collaboration, we delivered a pediatric critical care simulation curriculum for EMS providers that impacted participant knowledge and confidence caring for pediatric patients. Follow-up data suggest that knowledge and skills obtained as part of the curriculum was translated into practice. This strategy could be used in future efforts to integrate simulation into EMS practice.

## Introduction

In 2019, pediatric patients represented only 5.9% of all transports by emergency medical services (EMS) in the United States. (1) Prehospital encounters with critically ill children represent low frequency, high stakes events. In national estimates, <1% of pediatric transports involve interventions such as advanced airway management or cardiopulmonary resuscitation (CPR). (2) Yet EMS providers are an essential link in the chain of survival, and when needed, these pediatric skills must be executed effectively. Given limited clinical exposure, EMS providers cannot rely on experience alone to maintain proficiency in pediatric assessment and resuscitation skills and need access to effective continuing education opportunities (3, 4). Simulation has been demonstrated as an effective tool within medical education, including training for low frequency, high stakes events (5–7). However, simulation education can be resource intensive and costly. EMS providers face additional challenges when accessing pediatric simulation including lack of standardization in pediatric continuing education requirements, and limited access to subject matter experts and simulation resources (3). Research on the most effective means of pediatric continuing education for EMS providers is limited (3, 8–10).

The Emergency Medical Services for Children (EMSC) program at the federal and state level has the goal of reducing pediatric mortality and morbidity from severe illness and trauma (11). Collaboration between state EMSC programs and pediatric academic centers provides an opportunity to deliver accessible pediatric simulation to EMS providers and improve access to high quality education in pediatric resuscitation.

### Goals of This Investigation

Our aim was to use a statewide, multi-center collaboration to develop and deliver a pediatric critical care simulation curriculum for EMS providers. Our primary outcome was to evaluate the impact of the simulation program on participants' knowledge and confidence. We also report secondary outcomes including assessment of knowledge retention and assessment of the learners' perception of the curriculum.

## Materials and Methods

### Study Design

Through a statewide, multi-center collaboration, we developed and assessed a pediatric simulation curriculum for EMS providers. We then conducted a prospective, interventional, single-arm educational study with a pre-post assessment design to evaluate the impact on EMS provider knowledge and confidence. This study was deemed exempt by the Institutional Review Board.

### Study Population

The study population included licensed emergency medical technicians (EMTs) and paramedics in Massachusetts. EMTs are basic life support (BLS) providers with a minimum of 110 h required for initial certification. Paramedics are advanced life support (ALS) providers with between 1200–1800 h required for initial certification. Though Advanced Cardiac Life Support (ACLS) certification is required, Pediatric Advanced Life Support (PALS) certification is not mandatory for ALS providers in Massachusetts. Statewide, 62% of all 911 responses include an ALS provider, and 57% of pediatric responses include an ALS provider (12).

The simulation days were advertised through EMS region directors, on the state EMSC website, via social media, and through flyers distributed to hospital Emergency Departments and EMS agencies across the state. Grant funding through the Massachusetts Department of Public Health allowed participants to attend free of charge. For this study, we recruited a convenience sample of volunteer participants with efforts to recruit across all 5 EMS regions in the state. Study participation was voluntary. Participants who declined the pre- and post-assessment surveys were excluded from study data collection but still received the educational offering.

### Collaboration

The Massachusetts (MA) EMSC advisory committee includes physician faculty with expertise in pediatric emergency medicine (PEM) from five academic pediatric emergency departments who collaborated to develop and deliver a standardized pediatric critical care simulation curriculum. Each institution was paired with one of five geographically associated EMS regions. A physician faculty leader from each institution identified expert instructors and simulation resources within their organizations.

### Intervention: Curriculum Development

Content for simulation cases was chosen based on prior literature (2, 13), previously published EMS simulation scenarios (14), and the results of a statewide needs assessment which identified EMS provider discomfort caring for infants. The cases were all reviewed by the study team and faculty leaders. The cases were pilot tested locally with an agency not involved in the study and feedback was incorporated prior to implementation across all sites. The final versions of cases were reviewed with the EMS regional directors and EMS leaders on the EMSC committee.

Scenario design targeted assessment, clinical decision-making, practice of important life-support skills, and reinforced state EMS protocols. Key pediatric resuscitation skills included recognition of inadequate ventilation, airway positioning, bag-mask-ventilation, advanced airway management, IV and IO access, choosing appropriately sized equipment, and weight-based dosing of critical medications. Scenarios also integrated local patterns of EMS team composition and accommodated different scopes of practice. Cases were designed for BLS participants to “respond” to the scenario first to allow utilization of their resuscitation skills during initial management. In Massachusetts, some communities have BLS-only EMS services while some have a tiered response with BLS responding first and ALS intercepting as needed. For example, the twin field delivery, based on a previously published case (14), was structured so that the first baby responds to BLS resuscitation measures (high quality CPR and BLS airway management) while the second infant requires additional ALS resuscitation such as IO access, weight-based epinephrine, and intubation. The second scenario, based on a previously published case (14), adapted and piloted locally (15), required recognition of status epilepticus and inadequate ventilation requiring hands on practice with infant bag-mask ventilation as well as appropriate use of weight-based anti-epileptic medications. The third case was developed specifically to incorporate a recent statewide protocol change allowing BLS use of intramuscular (IM) epinephrine for asthma with impending respiratory failure.

Each participating institution used their own manikins with guidance provided regarding the size of manikin for each case (newborn, infant, and child) included in the facilitator guide. Examples of the high-fidelity simulators used included Gaumard Super Tory, Laerdal SimBaby, and Laerdal SimJunior. Each scenario included prescribed stages with vital sign ranges and exam findings as well as responses to several expected interventions. The manikin progression through the stages of the case and response to interventions were adjusted in real time using the case scenario templates to guide facilitators.

### Intervention: Curriculum Implementation

The pediatric critical care simulation curriculum included targeted learning objectives with standardized, structured debriefing. Each simulation was facilitated by a pediatric emergency physician, at times working with a simulation engineer, using high-fidelity manikins. Facilitators all had prior training in simulation and debriefing techniques including plus-delta and advocacy-inquiry (16). Faculty development training sessions were held by faculty leaders for facilitators to run through the cases and review key content for debriefing. All cases as well as a facilitator guide (Supplementary File 1), including summaries of operations and key content, were also distributed by email the week prior to each session. Facilitators met with simulation engineers on the day of each session to review the cases. The schedule was standardized across sites and distributed to facilitators the week prior to the sessions and to participants at the start of the day. In total, 34 pediatric emergency medicine physicians, 2 pediatric nurses, 5 paramedics, and 9 simulation engineers across 5 institutions delivered five 8-h, single-day pediatric education sessions.

For three regions, the simulation curriculum was held at a simulation center within the academic institution. For two regions, the simulation curriculum was held at a hotel or conference center and simulation equipment was brought on site. Each simulation room included an EMS “jump bag” with standard equipment as well as standardized medication, IV, and airway supplies. Grant funding allowed participants to attend free of charge and provided food for facilitators and participants. Facilitators not involved in the study received a small stipend for their participation.

To optimize facilitator-participant ratio and limit team size during simulation scenarios, the participants were divided in half, with one cohort starting with simulation and the other starting with didactic sessions. Participants rotated through 3 simulation rooms with one case and debrief in each room. Facilitators remained in the same scenario, while participants rotated through all three. The group that began with simulation then subsequently attended didactics and vice versa (Supplementary Figure 1). The didactic lectures included key concepts in pediatric care. Topics for didactic components of the program were not standardized across sites and, because of variability, content specific to the didactics was not included in the knowledge assessment.

### Measurements: Knowledge Assessment

Questions in the knowledge assessment were obtained from the National Registry EMT exam (17), a local pilot study (15), and a previously published EMS curriculum (14). The BLS knowledge assessment included 20 questions, the ALS assessment included an additional 5 questions specific to ALS scope of practice (Supplementary File 2). The knowledge assessment was limited to items pertaining to the simulation cases as these were standardized across all sites. The knowledge assessment was piloted with 15 EMS providers in Connecticut (CT) who were not part of the eligible Massachusetts study population, as well as five board certified pediatric emergency medicine (PEM) physicians to assess reliability and validity. To test reliability, 11 of the original 15 EMS providers completed the knowledge assessment again after 2 weeks without any additional pediatric training. The mean change in score was +3%. The intra-class correlation coefficient (ICC) was 0.74 (95% CI 0.31, 0.92). As an assessment of face validity, we compared the mean scores across the three groups. The PEM physicians had the highest mean score (98.4%), followed by the ten ALS providers (89%) and the five BLS providers (78%).

### Data Collection

Study participants completed the written knowledge assessment in person prior to beginning the curriculum (pre-test). At the conclusion of the curriculum, participants completed the identical knowledge assessment (post-test). Responses to pre- and post-tests were transcribed by research assistants into a REDCap (Research Electronic Data Capture) database hosted at Boston Children's Hospital using anonymous study ID numbers. REDCap is a secure, web-based software platform designed to support data capture for research studies (18, 19). Each participant's pre- and post-tests were linked to allow for analysis of change in test score. Attendance, and therefore our study sample size, was limited per site to maintain optimal group sizes for the simulation scenarios. Given our fixed sample size, we were adequately powered to detect a difference of at least 3% (or a 3 point difference in test score, measured from 0–100%) with a standard deviation of 10.

### Follow-Up Assessment

To assess knowledge retention, the identical assessment was administered between 5 and 6 months after the completion of the curriculum. Each participant received an email with a link to the follow-up assessment. Reminder emails were sent weekly for 4 weeks. In addition to the knowledge assessment questions, a follow-up survey included questions about any additional pediatric or simulation training participants had received since participation in the curriculum and whether they had applied knowledge or skills acquired during the simulation in practice. If participants responded yes, they were prompted to share an example of the knowledge or skill used in practice.

### Measure of Confidence

Participants reported their confidence caring for pediatric patients as part of the pre-curriculum, post-curriculum, and follow-up questionnaires. In the post-curriculum, participants were asked to estimate their change in confidence in caring for children with a scale of “less confident,” “no change,” and “more confident.” Percentage of participants who reported a positive change in confidence was calculated.

### Identifying Qualitative Themes

Study participants provided written comments at the conclusion of the simulation curriculum, evaluating the simulations, didactics, and facilities. During the follow-up assessment, participants provided comments regarding educational gains that were subsequently used in clinical practice. All free-text, qualitative comments were compiled in an Excel database (Microsoft Corporation. 2018) and independently analyzed for themes by 5 study team members using the framework method of qualitative analysis (20). One team member transcribed the comments verbatim. Three team members reviewed the comments and independently applied codes. Two team members reviewed the codes and independently assigned themes. Finally, two different team members, not part of any prior step, reviewed the proposed themes for consistency and consensus.

### Data Analysis

We characterized the demographic features of the participants, using frequencies with proportions and medians with interquartile ranges (IQR) for categorical and continuous variables, respectively. The pre-test and post-test for each participant was linked using participant ID numbers and the change in score was calculated. We calculated descriptive statistics within subgroups (BLS-only, ALS-only, agency type, EMS region, and years of clinical practice). The agency variable included fire-based, municipal, commercial, and other (including volunteer departments). EMS region was anonymized. Years of experience was dichotomized to ≤ 5 years or >5 years of clinical practice.

To assess the effect of the intervention on knowledge scores while accounting for the nested nature of the data (i.e., assessment nested within participants and participants nested within region), we estimated a mixed effects generalized regression model with random effects for region and participant, using the gamma family and log link. We modeled the knowledge score (percent correct) as the dependent variable and assessment time (pre-test, post-test, and follow-up) as the independent variable. We expressed differences in the knowledge score outcome between assessments as mean percent change with 95% confidence intervals (CIs).

To determine predictors of knowledge improvement, we estimated a multivariable generalized linear regression model using the binomial family and identity link. We modeled knowledge improvement (i.e., a delta score >0) as the dependent variable and agency type, region, BLS/ALS, and years' experience as the independent variables.

To examine associations between confidence improvement and participant characteristics, we categorized each participant as reporting a positive change in confidence vs. not. We tested associations between the binary confidence improvement variable and participant characteristics (agency type, region, BLS/ALS, and years' experience) using Fisher's exact test (to account for small sample sizes within cells). Alpha was set at 0.05 and all tests were two-tailed. Analyses were conducted using STATA MP Version 16.0 (Stata Corp, College Station, TX).

## Results

### Characteristics of Study Participants

**Figure 1:**
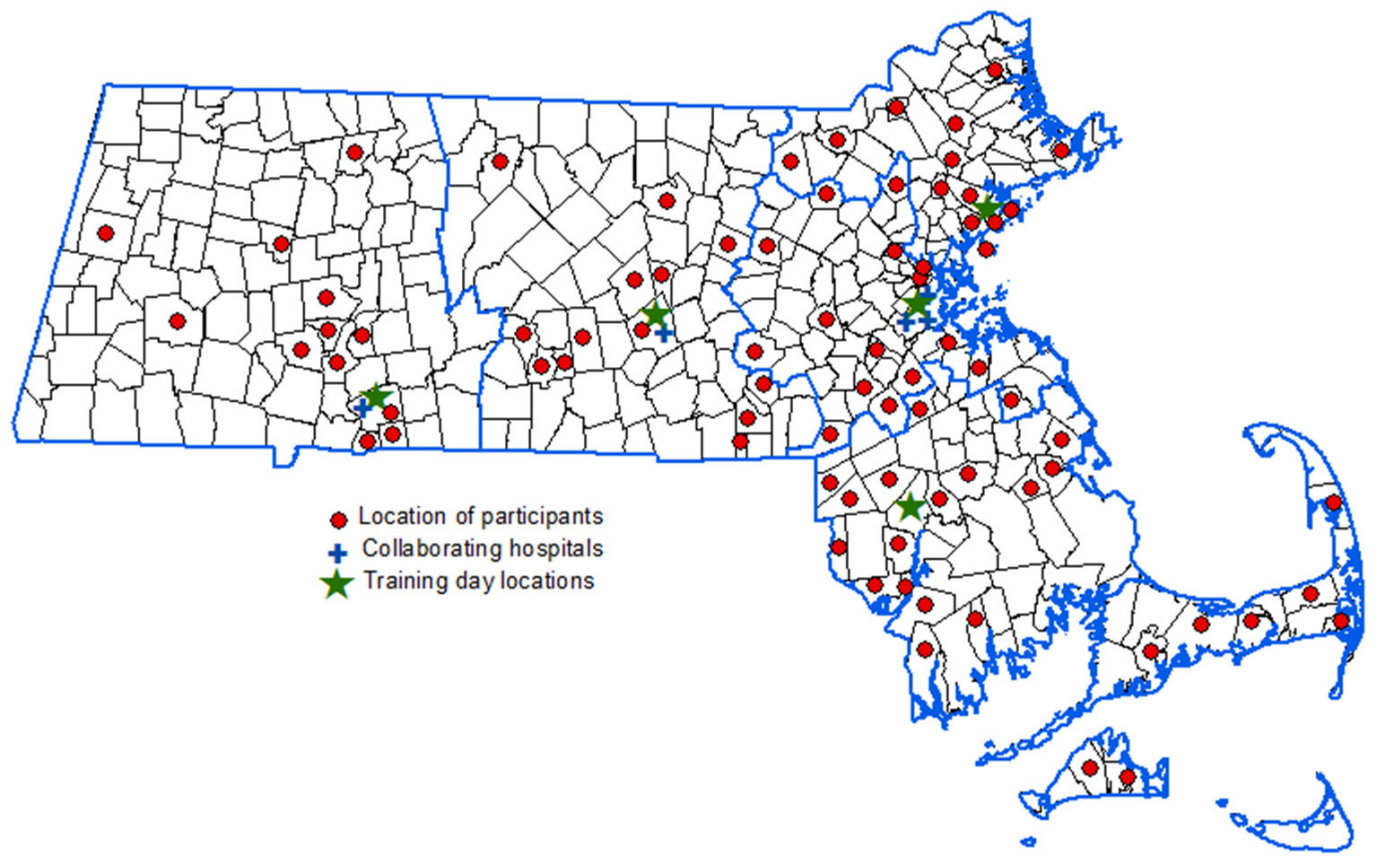
Distribution of participants across Massachusetts. Map of Massachusetts with the 5 EMS Regions outlined in blue. Red circles indicate towns and agencies represented by participants, blue crosses represent the participating hospitals, and the green stars denote the location of the educational sessions. The map was created using ArcGIS® software by Esri. ArcGIS® and ArcMap™ are the intellectual property of Esri and are used herein under license. Copyright^©^ Esri.

A total of 78 EMTs and 109 paramedics from the 5 EMS regions in Massachusetts, representing 97 different towns and EMS agencies (Figure 1), participated in the educational curriculum and completed both the pre- and post-curriculum assessments. The majority were ALS providers (58%) and male (69%). Participants had a range of prior field experience; from those with <1 year of experience (4%) to those with more than 15 years' experience (33%). The study population included fire-based, municipal, commercial, and volunteer EMS services (Table 1).

**Table 1:** Participant demographics.

	***n* = 187**
BLS*	78 (42)
ALS*	109 (58)
Sex (male)	116 (69)
Age (years)	
18–25	31 (18)
26–35	61 (36)
36–45	27 (16)
46–55	30 (18)
≥ 56	21 (12)
Years' experience	
0–1	7 (4)
2–5	55 (32)
6–10	30 (18)
11–15	22 (13)
≥ 15	56 (33)
Agency type	
Fire–based	55 (34)
Municipal	32 (20)
Commercial	45 (28)
^∧^Other	31 (19)
Region	
1	17 (9)
2	47 (25)
3	49 (26)
4	24 (13)
5	50 (27)
Service Area	
Rural	32 (19)
Suburban	69 (42)
Urban	44 (27)
Other/More than one	21 (13)
Service Profile	
Volunteer	7 (4)
Paid	142 (87)
Paid per call	11 (7)
>1 Service type	3 (2)

*Values in table represent frequency (proportion).*

*^∧^Includes volunteer services.*

^*^Basic Life Support (BLS) and Advances Life Support (ALS).

### Pre- vs. Post-curriculum Knowledge Assessment

The median pre-test scores were 60% (IQR 48–68%) for BLS providers and 80% (IQR 76–88%) for ALS providers. Both BLS and ALS providers demonstrated improvement in the post-curriculum knowledge assessment with a median change in score of 8% (IQR 0–12%) for BLS and 4% for ALS (IQR 0–12%) (Table 2). Sixty five percent of BLS providers and 65% of ALS providers demonstrated improvement on the post-test assessment. In the mixed effects regression model, there was statistically significant improvement in knowledge scores, with a mean 9.8% increase in knowledge scores in the post-test compared to the pre-test (95% CI: 7.2%, 12.4%). In the multivariable regression model with knowledge improvement (i.e., a delta score >0) as the dependent variable and ALS/BLS, agency type, binary years' experience and region as the independent variables, agency type and region emerged as significant predictors of improvement. Participants from municipal agencies were more likely to improve compared to both fire-based and commercial agencies.

**Table 2:** Knowledge assessment pre– and post–curriculum.

	***n***	**Baseline assessment (% correct)**	**Post–curriculum assessment (% correct)**	**Intra–Participant delta**
Total	187	72 [60, 84]	76 [68, 88]	4 [0, 12]
BLS	78	60 [48, 68]	64 [56, 72]	8 [0, 12]
ALS	109	80 [76, 88]	88 [80, 92]	4 [0, 12]
Agency Type				
Fire–based	55	76 [60, 84]	84 [64, 88]	4, [0, 08]
Municipal	32	74 [52, 82]	80 [60, 92]	8, [4, 12]
Commercial	45	72 [60, 84]	76 [68, 88]	4, [0, 12]
Other	31	72 [60, 80]	76 [68, 88]	8, [0, 12]
EMS experience				
0–5 years	62	60 [52, 76]	68 [60, 76]	4 [0, 12]
≥6 years	108	76 [64, 84]	84 [72, 92]	4 [0, 12]

*Values in table represent median percent correct and (interquartile range) is included in brackets*.

### Follow-Up Results

**Figure 2:**
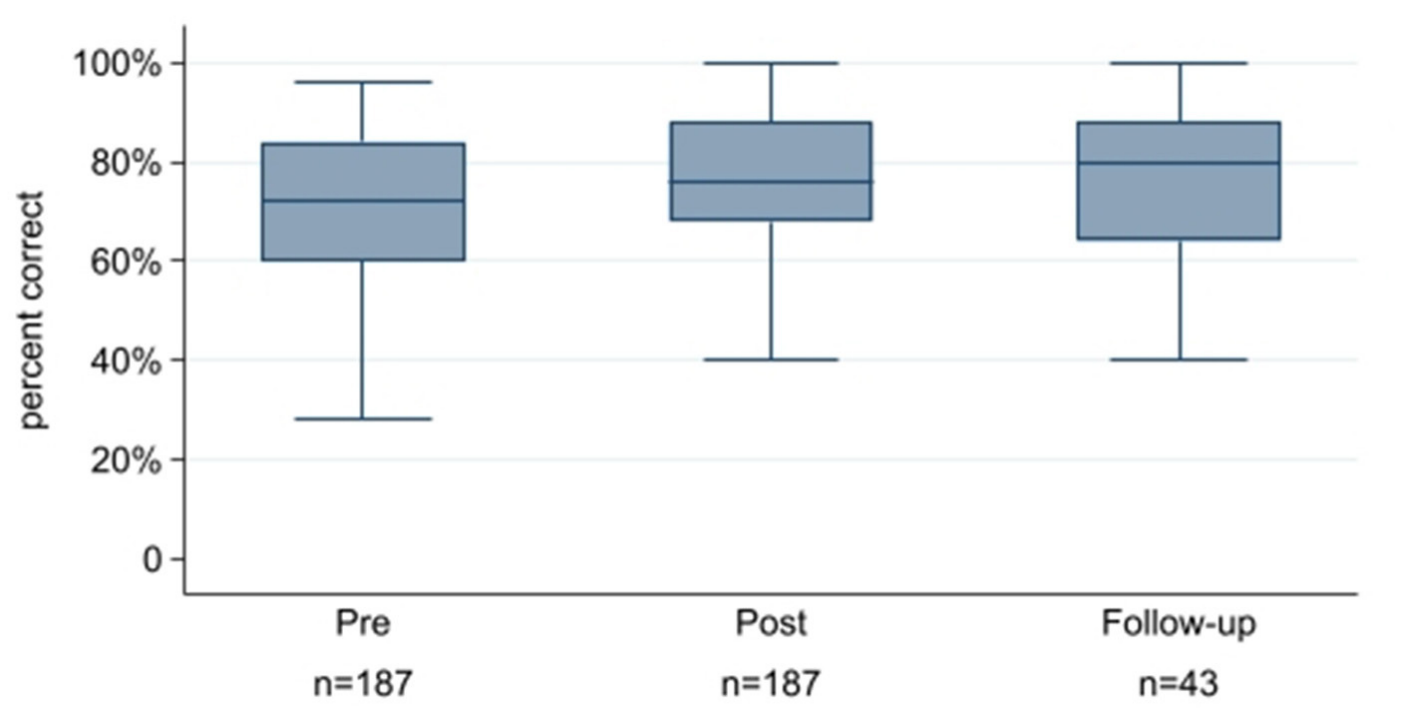
Knowledge change and retention at 6 month follow-up. Box plots represent scores on the knowledge assessment at the pre-training, post-training, and follow-up intervals. The lines are the median scores, the boxes are the 25th and 75th percentiles, and the whiskers are the upper and lower adjacent values.

Follow-up surveys were completed by 43 participants (23%). Those who completed the follow-up assessment were similar to the original study population in proportion of ALS providers (65%) but were significantly older and had more years' experience. Most respondents (88%) reported that they had no additional pediatric education and 72% reported no further simulation education since participating in the curriculum. Of those who completed follow-up assessments, 47% maintained a higher score at 6 months relative to their pre-test score (Figure 2) and 42% reported they had used knowledge or skills they learned during simulation in practice in the subsequent 6 months.

### Change in Confidence

During the pre-curriculum assessment, 39% of participants were “not comfortable” caring for newborns and 35% were “not comfortable” caring for infants. During the post-curriculum assessment, 92.8% of participants reported a positive change in confidence (95% CI: 87.2%, 96.5%). Using Fisher's exact test, there were no statistically significant associations between confidence improvement and ALS/BLS status, agency type, or years' experience.

### Qualitative Themes

Ninety participants (48%) included free text comments regarding participation in the simulation curriculum. Identified themes and representative comments are included in Table 3. Participants found the curriculum to be relevant, appreciated the interactive nature, found the opportunity for feedback and debriefing to be helpful, and identified chances to collaborate and work as a team (Table 3). Participants, especially during simulation days that were full to capacity, noted crowding and recognized potential benefit to having smaller simulation groups in the future. Of the 43 participants who completed 6-month follow-up assessments, 19 provided data regarding use of skills learned during simulation in clinical practice (assessment and approach to pediatric patients and interventions) as well as feedback on their confidence caring for children and approach to education following the curriculum (Table 3).

**Table 3:** Qualitative themes identified.

**Themes**	**Representative quotes**
**Post**–**training survey**	
Feedback/debriefing	“Feedback from physicians was really helpful. So often we treat and transport and hope we did well. Feedback today verified that we do. Incredible training. Thank you.” “It was great to get feedback from ER docs for real life scenarios. I was able to clarify many areas that I was uncomfortable with before.”
Relevant content	“Useful skills demonstrated, clearly explained, used good examples, a review of basic skills. I learned about new updates for medications for basic EMT protocols. Very helpful. A worthwhile event for all.” “Pedi is a complex, low volume call so constant re–ed is a plus. Liked the mix of sim with review.”
Interactive	“The manikins are an invaluable tool to learn from. Very well presented. Very engaging.” “Seizure case in particular– Great scenario to get you thinking. Excellent learning opportunity, excellent course.”
Collaboration/teamwork	“Awesome to work with other providers who I'm not comfortable working with.” “BLS first response was very helpful and excellent for continuity of care.”
**6**–**month follow**–**up survey**	
Assessment	“My patient assessments are more focused. I am more organized in my assessments, finding and noting abnormalities or norms. I give a better report/hand–off.” “Better assessment skills with pediatrics and interfacing with parents.”
Approach	“Newborn care after field birth.” “The seminar was great attending because we do not have many interactions with infants or children. It was awesome to help refresh us.”
Intervention	“We ran a severe asthma pediatric pt, the week after the class. I credit the class with my ability to recognize and treat as aggressively as needed.” “Epi Pen Jr for an infant with severe respiratory distress and wheezing. Implement better care for an infant experiencing a seizure.”
Confidence	“Much of the knowledge gained from the Sim course, I hope I never ever have to use. That being said, being able to practice these skills in the lab was very helpful, and will make me a more effective provider in the situation I have a critical pedi Pt.” “The training gave me more confidence working with pediatric patients.”
Education	“I am a paramedic clinical educator. We use simulation from time to time but we're renewed in our mission to offer more simulation to our EMT's and Medics.” “Given the stressful nature of younger critical pts, its always useful to drill through scenarios with the quality of manikins we had to use. During those stressful times, providers rely on the quality of training they've been exposed to in combination with experience of past cases, to rationally and calmly identify and treat critical illness. I wish more pushed themselves and weren't afraid to make mistakes in environments like this.”

## Discussion

Ensuring EMS providers are prepared to care for critically ill children is a challenge due to limited access to pediatric education and low volume of critically ill pediatric patients nationally. Though they represent an essential link in the chain of survival, EMS providers often have limited pediatric training opportunities and can be overlooked in efforts to improve pediatric resuscitation. Through this study, we demonstrate EMSC partnerships as one strategy to provide access to pediatric simulation with potential for wide reaching impact. Our simulation curriculum for pediatric critical care resulted in statistically significant knowledge gains. In addition, 93% of participants reported increased confidence caring for pediatric patients after this intervention. Though more challenging to measure, participant self-report during follow-up assessment suggest participation in simulation influenced their real world performance.

### Pediatric Simulation and EMS

EMS providers desire increased pediatric education; specifically, experiences utilizing hands-on training, including high fidelity simulation (3, 13, 21). Opportunity for real-time feedback to modify performance as well as post-scenario debriefing facilitates learning and participants recognized this benefit from participating in our curriculum. Prior work has demonstrated simulation with facilitated debrief uncovered causes of errors in pediatric care by EMS providers (22). The National Council of State Emergency Medical Services Training Coordinators recommends regular review of pediatric skills without reliance on clinical exposure as a measure of competency (23). Current EMSC performance measures set a target of at least 60% of all EMS agencies will have a process that requires EMS providers to physically demonstrate correct use of pediatric specific equipment by 2023 (24). A simulation curriculum such as the one we developed provides an opportunity for such demonstration.

Baseline knowledge scores before participation in the curriculum highlight room for improvement in pediatric knowledge, especially among BLS providers. Though knowledge gains were small, they were statistically significant and 65% of participants demonstrated improvement. Prior studies have suggested that practicing a procedure during simulation improves clinical practice performance (5, 8, 25). Previous work has demonstrated improved adherence to pediatric EMS protocols and improved outcomes after participation in a simulation curriculum (26, 27). Our multi-center study demonstrates the ability to reach and potentially impact a broad audience across the state. The challenge in educational interventions remains to determine what measure of knowledge, experience, and confidence translates to a measurable improvement in clinical practice, which is beyond the scope of this study. We believe our work adds a potential scalable model for dissemination of pediatric simulation for EMS. We were able to mitigate previously identified barriers to bring pediatric simulation to wide-scale EMS audiences. Recognizing the benefit of simulation and facilitated debrief found in prior work (22), a model like ours provides the opportunity for larger groups of EMS providers to have those experiences facilitated by their local PEM experts.

### Confidence

In addition to changes in knowledge, EMS providers also improved their confidence in caring for children. It is important to recognize that participant confidence does not equate with competence and available data suggests that health care providers, including paramedics, are not always accurate in their self-assessments (28–30). Yet, it is also important to recognize that EMS providers identify stress and anxiety around pediatric care as a major contributor to patient safety events (31). Prior simulation work has demonstrated improved performance with improved confidence (32, 33). However, in our study, using Kirkpatrick's 4-level training evaluation model (34), we chose to only assess the first two levels of impact, learners' reactions and learning (improvement in knowledge). We therefore cannot comment on how improved confidence translated into changes in clinical care in our study population. This collaboration is a first step toward additional multi-center work to use simulation as both an educational and evaluative tool to better understand the relationship between self-efficacy and skill performance.

### Statewide Collaboration

Despite the benefits of simulation, there is a paucity of this type of pediatric education for EMS providers (21, 35). Identified barriers including lack of funding, lack of access to pediatric experts, and lack of continuing education dedicated to pediatrics (36). We were able to mitigate some of these challenges through statewide collaboration which allowed us to bring the resources of academic medical centers, including simulation equipment, pediatric specialists, and simulation experts from across the state, to the prehospital providers who may not be able to access these resources independently. Shared responsibility across the 5 academic centers resulted in delivery of the standardized simulation curriculum 5 times over 6 months without undue burden on any one hospital or simulation program. Lastly, the collaboration provided an opportunity for communication and relationship building. EMS providers debriefed simulated cases with their local PEM physicians whom they might normally only see in an actual pediatric transport.

### Innovation and Lessons Learned

The multisite approach we employed is an innovative method to allow for a scalable regional model. Our experience provided insight for others who may want to deliver a similar program. Including broad input from multidisciplinary stakeholders and EMSC committee members during the planning of this curriculum, as well as standardizing the delivery, likely contributed to this success. Additionally, the importance of a central lead to coordinate such a large undertaking was a significant factor for success. A faculty lead for each site was also essential to coordinate recruitment of speakers and facilitators, review cases with facilitators, and coordinate simulation resources. While the curriculum remained constant, scheduling the sessions across the state over a 6 month time period allowed us to improve the logistical process with each iteration with participant recruitment, registration, and flow through the day enhanced in subsequent sessions. The faculty leads for each site met (either via phone or in person) after each of the 3 first sessions to debrief and share lessons learned and logistical tips for the subsequent sessions.

The collaboration between pediatric academic centers and the MA EMSC remains strong. While plans for additional in-person simulation training were paused due to the COVID-19 pandemic, we currently have plans underway to bring ongoing pediatric training to the MA EMS community.

### Generalizability

Given knowledge improvements across the 5 EMS regions, we believe this model of a shared curriculum delivered by different instructors and with different manikins is generalizable and reproducible, and others may benefit from similar collaborations despite potential differences in state size, population density, or EMS provider distribution. Though there were differences in baseline knowledge across the EMS regions, there were similar knowledge improvements at the end of the curriculum. Both ALS and BLS providers were able to demonstrate knowledge gains, suggesting that the included cases effectively met the needs of learners with a wide range of prior experience and knowledge. The differential margin of improvement between BLS and ALS providers is informative for future education as close attention is needed to ensure adequate BLS learning objectives and consideration of the BLS/ALS distribution across sites.

Finally, this collaboration among academic institutions has established the groundwork for future continuing education of EMS providers in our state. Such inroads are important for sustainability of such efforts. We recognize our state is well-positioned for such a collaboration given the presence of several academic pediatric emergency departments. While other states may not have the same distribution of pediatric resources, partnership between state EMSC programs and general emergency departments may represent another option for pediatric education.

### Limitations

This study was designed only to evaluate knowledge and confidence, therefore, we are unable to assess for an associated impact on clinical outcomes. Additionally, we do not know if these modest improvements in knowledge will translate to clinical care or outcomes. By using a multiple choice assessment tool, we are limited in our ability to measure the constructs of skill improvement. A more robust assessment option could have included pre-post assessment using simulated scenarios. However, this would have added additional time and cost to the curriculum that was prohibitive at the time. Alternative modalities for assessment should be considered in future work. Additionally, in order to provide pediatric critical care education to as many EMS providers as we could with our funding, we did not design this study with a control arm. This limits the ability to compare our simulation curriculum against a less expensive pediatric education modality. Recent work suggests that less expensive, low-fidelity simulation may be adequate for improved performance in pediatric education for paramedics which we will consider for future efforts (37). Our qualitative results do suggest the interactive cases and real-time feedback from facilitators were important to participants. However, having multiple study team members participate in different stages of the qualitative assessment is a potential source of bias. The assessment of the longer-term educational benefit was limited by relatively low participation in the 6-month follow-up despite voluntary participation in the curriculum and provision of contact information for follow-up. This speaks to the difficulty of demonstrating sustained benefit and is an important consideration for future studies. Several studies have demonstrated knowledge attrition occurs after initial educational interventions, suggesting that frequent educational interventions are helpful for maintenance of educational benefits. (10, 38, 39) Additionally, this curriculum was not mandatory, and participants represented a small proportion of EMS providers in Massachusetts. Those who participated may be highly motivated learners or particularly interested in pediatrics and therefore more likely to benefit. Furthermore, in efforts to optimize the simulation team size, we had participants rotate out of simulation and through didactics. With a captive EMS audience, we chose to provide pediatric didactics in addition rather than run half-day simulations, given the limited opportunities to access pediatric education. In order to provide flexibility for faculty participation, there was variability in didactic topics across sites. This variability may have contributed to changes in knowledge. We attempted to mitigate any impact of this site-level variability by not including content specific to didactics in the knowledge assessment. The knowledge assessment was limited only to the simulation content which was standardized across all sessions. However, we recognize this may have introduced variability in the overall education delivered and in the future will standardize didactics to support simulation learning objectives. Lastly, and perhaps most importantly to those seeking to replicate this effort, this was a funded effort. Grant support covered the cost of simulation, all supplies, as well as meals for participants. The total cost of this effort, including food and administrative fees, was $80,000 over 6 months. Cost varied by site with lowest cost at sites able to host at an on-site simulation center ($8,000) and highest cost when a hotel conference center was used ($25,000). While the cost of high-fidelity simulation limits it accessibility for individual towns and EMS agencies, the cost remains a challenge for state agencies and academic partnerships. This has implications for the feasibility of other efforts to deliver simulation education.

## Conclusions

Through EMSC partnership and statewide collaboration, we were able to pool our resources to develop and deliver a pediatric critical care simulation curriculum for EMS providers. The impact of participation included modest improvements in knowledge assessment scores and increased confidence in caring for pediatric patients. We did not directly study clinical performance however follow-up responses suggested some participants were able to translate this experience into practice change. While future work is needed to ensure sustainability and to reach even larger populations of EMS providers, this collaboration can serve as the foundation for future pediatric education for EMS providers, an essential link in the chain of survival.

## Data Availability Statement

The raw data supporting the conclusions of this article will be made available by the authors, without undue reservation.

## Author Contributions

CF, KD, JN, and SC conceived the study, designed the trial, and obtained research funding. CF, KD, BM, TB, AK, JD, JL, BW, JN, and SC supervised the conduct of the trial and data collection. CF, JD, and MM managed the data and including quality control. MM provided statistical advice on study design and analyzed the data. CF, KD, BM, TB, JL, JN, and SC drafted the manuscript. CF takes responsibility for the paper as a whole. All authors contributed substantially to its revision. All authors contributed to the article and approved the submitted version.

## Funding

This education series was developed, in part, under grant number SM062910 from SAMHSA. It was administered by the Massachusetts Emergency Medical Services for Children state program. The views, opinions and contents of this publication are those of the authors and contributors, and do not necessarily reflect the views, opinions, or policies of CMHS, SAMHSA, or HHS, and should not be construed as such. CF did not receive funding related to this manuscript, but did received a philanthropic grant (Boston Strong Emergency Preparedness Fund) which has supported local efforts for pediatric prehospital readiness. TB did not receive funding related to this manuscript, but is funded by the NHLBI (1K23 HL 145126–01A1) for work to improve prehospital pediatric emergency readiness with telemedicine. SC did not receive funding related to this manuscript, but is the Co–director of Disaster Domain Emergency Medical Services for Children Innovation and Improvement Center and received grant support as the site PI for the Massachusetts/Region 1 Partnership for Regional Health Disaster Response US Department of Health and Human Services, Assistant Secretary for Preparedness and Response (1HITEP180042–01–00 and 6 HITEP180042–01–05 Site PI).

## Conflict of Interest

The authors declare that the research was conducted in the absence of any commercial or financial relationships that could be construed as a potential conflict of interest.

## Publisher's Note

All claims expressed in this article are solely those of the authors and do not necessarily represent those of their affiliated organizations, or those of the publisher, the editors and the reviewers. Any product that may be evaluated in this article, or claim that may be made by its manufacturer, is not guaranteed or endorsed by the publisher.

## Supplementary Material

Supplementary File 1 Facilitator's guide (includes introduction, schedule, and 3 simulation scenarios with debriefing guide).

Supplementary File 2 Knowledge assessment.

Supplementary Figure 1 Schedule of the day.
