# Supplementary material for: A Statewide Collaboration to Deliver and Evaluate a Pediatric Critical Care Simulation Curriculum for Emergency Medical Services
Source: Front Pediatr. 2022 Jun 14;10:903950. doi: 10.3389/fped.2022.903950 (PMC9237480; doi:10.3389/fped.2022.903950)
Supplement: Supplementary File 1 — Facilitator's guide (includes introduction, schedule, and 3 simulation scenarios with debriefing guide). [file Data_Sheet_1.PDF]

EMSC Pediatric Simulation 2019 Facilitator Guide

Table of contents

Introduction to EMS ……………………………………………………………………………………….2

Schedule of Events 3

Facilitator Roles 4

Simulation 1: The Seizing Infant (30min) 5

Case Progression……..…………………………………………………………………...5
Debriefing Points ……………………….…………………………………………........…8
BLS-only Version…………………………………………………………………………9
BLS-only Debrief…………………………………………………………………………..12

Simulation 2: The Critically Ill Asthmatic (30min) 13

Case Progression…………………………………………………………………………13
Debriefing Points 16

BLS-only Version…………………………………………………………………….…17
BLS-only Debrief ……………………………………………………………………………20

Simulation 3: Newborn Resuscitation (30min) 21

Case Progression 21
Debriefing Points 26

Weight Based Dosing per EMS Protocols………………………………………………..…28

EMS Protocols for reference …………………………………………………………………………
Seizure
Asthma
Newborn Care
Newborn Resuscitation

Brief Introduction to EMS

Emergency Medical Services are provided by Emergency Medical Technicians (EMTs). In Massachusetts, there are EMT-Basics (EMT-B) and EMT-paramedics (EMT-P). For the purposes of this course, we will refer to EMT-Bs as EMTs and refer to paramedics as paramedics. EMTs provide Basic Life Support (BLS) services. Paramedics provide Advanced Life Support services. EMTs and paramedics receive proportionally less pediatric training for their initial certification. There is also less required pediatric continuing education to maintain their certification annually. Pediatric 911 calls make up approx. 6-7% of all EMS responses in Massachusetts.

BLS services include:

- Newborn delivery
- Hemorrhage control
- PO glucose administration
- Extrications
- Basic burn management
- Stabilize and splint injuries
- Open and maintain airway
  - Supplemental O2, bag mask ventilation
- Boston EMS-specific protocols
  - BLS providers can give albuterol via neb to a known asthmatic
  - BLS providers can give IM epi to a known asthmatic in extremis

ALS services include:

- Advanced newborn resuscitation
- Intubation, including RSI
  - Surgical airway, pleural decompression
  - CPAP
- 12 lead EKG interpretation
- Manage infusions
- Pharmacologic interventions
  - ACLS meds, narcotics, benzodiazepines

******This will likely be the first simulation experience for most of the participants******

-Remind them of a safe space to make mistakes and learn.

-You may need to encourage them to actually TOUCH the mannequin and actually DO their assessments and treatments.

Schedule of Events

	Group A			Group B		
**0800**	Registration/Breakfast/Begin pre-assessment					
**0830**	Welcome Message from EMSC					
**0910-0955**	Keynote Speaker					
**1000-1130**	Sim 1<br>30 minutes<br>Seizure	Sim 2<br>30 minutes<br>Asthma	Sim 3<br>30 minutes<br>Newborn	Didactic<br>45 minutes	Didactic<br>45 minutes	
**1130-1145**	Break					
**1145-1230**	Lunch (with Didactic)					
**1245-1415**	Didactic 1<br>45 minutes	Didactic 2<br>45 minutes		Sim 1<br>30 minutes<br>Seizure	Sim 2<br>30 minutes<br>Asthma	Sim 3<br>30 minutes<br>Newborn
**1415-1430**	Break					
**1430-1515**	Panel Discussion (PEM Staff) moderated by EMSC					
**1515-1545**	Feedback and post-assessment					

Facilitator Roles

- Participants will be divided into **10 person (max)** groups with **2 facilitators**.
    - Participating Facilitator- to provide history, answer questions on parents' behalf
    - Observing facilitator- if available, to take notes for debriefing

- Each group will have **both ALS and BLS providers.**
    - Each group will have a mix of EMT Basics (BLS) and paramedics (ALS).
    - There is a "BLS Only" version of each case in case you have crews of BLS-only providers
    - EMTs and paramedics will alternate so that all 10 team members have the opportunity to have hands on the manikin during at least 1 of the 3 simulations.
    - Up to 4 EMTs/paramedics can participate in any 1 scenario, the rest of the team should observe.
    - All 10 team members should participate in the debriefing

- Each simulation will be **a total of 30 minutes**.
    - The scenario should conclude by 10 minutes (15min max).
    - There should be 15-20 minutes for debriefing after the case.

DURING the simulation:

- Provide additional history if asked.
- Redirect the crews if they report or act on something that is manikin artifact.
- Redirect the crews if they are missing a key exam or history finding to guide the case.
    - For example, if the crew does not recognize apnea or seizure activity, suggest "*I think the child's breathing has changed*" or *"I think the baby started seizing again."*

******This will likely be the first simulation experience for most of the participants******
-Remind them of a safe space to make mistakes and learn.
-You may need to encourage them to actually TOUCH the mannequin and actually DO their assessments and treatments.

Scenario Title:	Simulation 1: Infant seizure<br>**ALS or mixed ALS/BLS VERSION**	Author:	Caitlin Farrell
Department:	Medicine	Division:	Emergency Medicine

Learning Objectives:

By the end of this session, participants should be able to:

- Assess an infant (actively seizing and post-ictal)
- Recognize signs of inadequate ventilation (snoring, slow RR, elevated ETCO2)
- Manage the airway in a post-ictal infant (reposition, OPA/NPA, O2, end tidal)
- Recognize acute change in mental status/VS and recognize seizure activity
- Appropriately treat GTC in an infant: check blood glucose, support airway, administer IN midazolam.
- Recognize risk for hypoventilation following benzodiazepines

Patient Information:

Name	Samantha Jones	Age	4mo
Dx	Seizure	Gender	F
Sx	GTC, 5min, self-resolved	Weight	5kg (11lbs)
MRN		Allergies	NKDA

Patient History / Background:

"You are first to arrive on scene of a 4mo ex-28wk F. Mom called 911 and reports Pt had a GTC seizure which lasted about 5 minutes. Given her medical complexity and history of going into status, ALS has also been dispatched and is en route. Mother is here and can answer questions. Your equipment is here (point to BEMS jump bag)."

In pilot testing, there was discussion that ALS would NOT routinely be dispatched for a seizure as most pedi seizure calls are febrile seizures. Just move past this, the ALS crew is coming so they can all learn from the case together.

Additional History if asked:
-Has had GTC seizures in the past, but none recently since starting Keppra.
-Missed last 2 doses of Keppra.
-No fever.
-No known trauma.
-No vomiting, no diarrhea.
-Normal POs. Normal UOP.

PMH: *Ex-28wk, prolonged NICU course for respiratory issues.*
Only current medical problem is seizures.

NKDA, Immunizations UTD, only medication is keppra

Simulator(s)		Monitors		Initial Mannequin Props / Set Up:	
Neonatal		EKG		IV/ART Access:	No access to start.<br>*EMS can place line using their equipment*
Infant	X	NIBP			
Pediatric		SpO2		Resp Equipment	None on to start.
Adult		RR			
		ETCO2		Medications	Midazolam (IV/IO/IN)
**To be filled out by SIMPeds**		Temp			
NewBornHAL		ABP		Fluids	IVF
PediHAL		CVP			

Tory		ICP		Props / Moulage	G-tube taped to skin, will need equipment if they want to vent GT<br>-60cc syringe<br>-Tubing
SimBaby		LAP/RAP			
SimJunior		Others		Equipment needed during simulation	
SimMan		Would you like all monitors to be initially on?<br><br>☐Yes ■o		EMS equipment:<br>-IV/IO access<br>-BVM, non-rebreather, end tidal monitor, NPA/OPA<br>-Infant laryngoscope, ETTs, end tidal<br>-Glucometer	
SimMan3G					
SimManEssential					

**State 1**	Post-ictal on arrival		Estimated Time	(2min)
Vitals		Assessment / Details	Expected Interventions	
**HR**	130	Pupils: PERRL	**Expectation #1:** Assess infant and recognize post-ictal state.<br>**Expectation #2:** Recognize inadequate respiratory effort.<br><br>**Goals:**<br>-Reposition airway or place OPA/NPA or shoulder roll<br>-Apply supplemental O2<br>-Can assist ventilations w/ BVM	
**BP**	90/50	*Lung Sounds: Snoring respirations. No stridor, no wheezing.*		
**SpO2**	92%	*Heart Sounds: Normal*		
**RR**	10	*Not seizing. Post-ictal state on EMS arrival*		
**CVP**		*Verbal Response: Unresponsive/moaning*<br><br>*If asked, skin color is pink, warm, NOT FEBRILE*		
**ETCO2**				
**OTHER**				
Notes for Specialist:			**Facilitator Notes:**	
Not crying, just moaning or snoring noise if anything.<br><br><br>-If no intervention to support respirations, can ask Sim engineer to drop pulse ox to 80s and make baby cyanotic.<br>-If still not intervention to support breathing, then just move to State #2 and begin seizing.			**BLS arrives on scene first** and begins assessment.<br>-If time is passing and they do not recognize inadequate respirations, you can prompt as Mother- **"It looks like she's not breathing right"**<br>-If no intervention to support respirations, can ask Sim engineer to drop pulse ox to 80s and make baby cyanotic.<br>-If still not intervention to support breathing, then just move to State #2 and begin seizing.<br>****Prompt them for ALS arrival before the infant begins seizing again. If she's seizing with BLS-only crew, they will (appropriately) scoop and run****	

**State 2**	Actively Seizing	Estimated Time	5min
Vitals	Assessment / Details	Expected Interventions	

**HR**	Abruptly tachycardic to 180	Pupils: PERRL, maybe constricted from prior	* **Expectation #1:** Recognize acute change in VS (will only be on the monitor once ALS arrives and places Pt on monitor)<br>* **Expectation #2:** Recognize GTC seizure.<br><br>**Goals:**<br>-Recognize seizure<br>-Recognize apnea and give BMV<br>-Check bG (Result 90)<br>-Administer dose-appropriate midazolam (IN/IV)<br>-Consider IV access.	
**BP**	120/60	*Lung Sounds: Apneic. Perioral cyanosis*		
**SpO2**	Loss of tracing	*Heart Sounds: Tachycardic*		
**RR**	Apneic	*Seizure: Generalized tonic-clonic seizure.*		
**CVP**		*Verbal Response: None.*		
**ETCO2**				
**OTHER**				
Notes for Specialist:			**Facilitator Notes:**	
Patient will not be on the monitor until ALS arrives and places on monitor.<br><br>Seizure should continue until midazolam is given. Can stop as soon as midazolam is given and move to State 3.			Prompt ALS team to join if they have not at this point.<br><br>Prompt "It looks like the baby isn't breathing right" or "It looks like the baby is making funny movements, etc" if they do not notice the change.<br><br>Have seizure continue until midazolam is given.	
**State 3**	Post-ictal after midazolam administration		Estimated Time	(3min)
Vitals		Assessment / Details	Expected Interventions	
**HR**	130	Pupils: PERRL	* **Expectation #1:** Recognize post-ictal state<br>* **Expectation #2:** Recognize risk for hypoventilation while post-ictal and when post-benzos<br><br>-Will need to support ventilation with either BVM or reposition/OPA and reassess<br>-Goal to have Pt on end tidal CO2 for transport	
**BP**	90/50	*Lung Sounds: Clear to auscultation*<br>**Irregular respiratory effort, snoring sounds*		
**SpO2**	91% if no O2, 98% if on O2	*Heart Sounds: Tachycardic, no murmur, no gallop.*		
**RR**	10	*Seizure: No seizure. Decreased tone (will need to describe)*		
**CVP**		*Verbal Response: Withdraws to pain.*		
**ETCO2**	55			
**OTHER**				
Notes for Specialist:			**Facilitator Notes:**	
			Prompt to recognize hypoventilation: "It sounds like the baby is snoring." "Her breathing looks irregular"<br>Goal is not to intubate, just recognize and support hypoventilation.<br><br>Can end scenario by, "Fast forward a few minutes and you've arrived at the ED."	

	-Have them give report and use that as jumping off point for debrief.

<u>Debrief Points: Infant Seizure</u>
(Tailor to each crew based on what comes up during case)

1) Discuss risk of and promote recognition of hypoventilation in the post-ictal and post-benzo state

2) BLS- importance of positioning to maintain open airway when post-ictal
Chance to re-iterate importance of positioning to effectively ventilate an infant

3) Use of end-tidal CO2 in post-ictal child, monitoring for inadequate ventilation
**Lots of patients arrive to ED post-ictal, 100% on non-rebreather, but when placed on end tidal are >70!*
**Importance of monitoring vital signs en route to hospital to recognize return of subtle seizure activity (eye deviation, tachycardia, hypoventilation)*

4) Acknowledge use of other benzos (IM/IV Ativan, IM/IV midaz) and will have weight based doses in facilitator guide), but per MA EMS protocol, IN midaz is first line.

If rapidly proceed through case, can expand debriefing to talk about next steps:
- -Management en route, safe positioning for transport and monitoring
- -Effective BVM monitoring for chest rise
- -Airway management
- -Additional doses of benzodiazepines
- -Can consider waiting out the benzo effective if you are effectively bagging

Unlikely to proceed to intubation given time frame of scenario, but can discuss “infant intubation pearls” in debrief if needed:
-Infants will desaturate quickly, pre-oxygenate as able
-Review choosing blade and tube size for age

<u>EMS RSI Waiver: (This protocol has been updated to include rocuronium, but this list as of 2019 curriculum)</u>
<u>*There are only a few cities in MA with have RSI waivers.</u>
(Less than 30 kg):
Fentanyl 1-2 mcg/kg and / or versed 0.05-0.1 mg/kg
Atropine 0.01mg/kg IV (Pt less than 30 kg)
Etomidate 0.2-0.3 mg/kg MAX 20mg - only in children >10 yr old

**Scenario Title:**	**Simulation 1: Infant seizure**<br>****BLS ONLY VERSION****	**Author:**	

**Department:**	Medicine	**Division:**	Emergency Medicine

**Learning Objectives:**	By the end of this session, participants should be able to:<br>• Assess an infant (actively seizing and post-ictal)<br>• Recognize signs of inadequate ventilation (snoring, slow RR)<br>• Manage the airway in a post-ictal infant (reposition, OPA/NPA, O2)<br>• Recognize acute change in mental status/VS and recognize seizure activity<br>• Recognize risk for hypoventilation while post-ictal

**Patient Information:**	**Name**	Samantha Jones	**Age**	4mo
	**Dx**	Seizure	**Gender**	F
	**Sx**	GTC, 5min, self-resolved	**Weight**	5kg (11lbs)
	MRN		**Allergies**	NKDA

**Patient History / Background:**	***"You are first to arrive on scene of a 4mo ex-28wk F. Mom called 911 and reports Pt had a GTC seizure which lasted about 5 minutes. Given her medical complexity and history of going into status, ALS has also been dispatched and is en route. Mother is here and can answer questions. Your equipment is here (point to BEMS jump bag)."***<br><br>***In pilot testing, there was discussion that ALS would NOT routinely be dispatched for a seizure as most pedi seizure calls are febrile seizures. Just move past this, ALS is coming so they can all learn from the case together.***<br><br>*<u>Additional History if asked:</u>*<br>*-Has had GTC seizures in the past, but none recently since starting Keppra.*<br>*-Missed last 2 doses of Keppra.*<br>*-No fever.*<br>*-No known trauma.*<br>*-No vomiting, no diarrhea.*<br>*-Normal POs. Normal UOP.*<br><br>***PMH:*** *Ex-28wk, prolonged NICU course for respiratory issues.*<br>*Only current medical problem is seizures.*<br><br>*NKDA, Immunizations UTD, only medication is Keppra*

**Simulator(s)**		**Monitors**		**Initial Mannequin Props / Set Up:**	
Neonatal		EKG		IV/ART Access:	No access to start.
Infant	X	NIBP			
Pediatric		SpO2		Resp Equipment	None on to start.
Adult		RR			
		ETCO2		Medications	
**To be filled out by SIMPeds**		Temp			
NewBornHAL		ABP		Fluids	
PediHAL		CVP			
Tory		ICP		Props / Moulage	G-tube taped to skin, will need equipment if they want to vent GT
SimBaby		LAP/RAP			

					-60cc syringe<br>-Tubing
SimJunior		Others		Equipment needed during simulation	
SimMan		Would you like all monitors to be initially on?<br><br>☐Yes ■o		EMS equipment:<br>-BVM, non-rebreather, NPA/OPA	
SimMan3G					
SimManEssential					

**State 1**	Post-ictal on arrival		Estimated Time	(2min)
Vitals		Assessment / Details	Expected Interventions	
**HR**	130	Pupils: PERRL	**Expectation #1:** Assess infant and recognize post-ictal state.<br>**Expectation #2:** Recognize inadequate respiratory effort.<br><br>**Goals:**<br>-Reposition airway or place OPA/NPA or shoulder roll<br>-Apply supplemental O2<br>-Can assist ventilations w/ BVM	
**BP**	90/50	*Lung Sounds: Snoring respirations. No stridor, no wheezing.*		
**SpO2**	92%	*Heart Sounds: Normal*		
**RR**	10	*Not seizing. Post-ictal state on EMS arrival*		
**CVP**		*Verbal Response: Unresponsive/moaning*<br><br>*If asked, skin color is pink, warm, NOT FEBRILE*		
**ETCO2**				
**OTHER**				
Notes for Specialist:			**Facilitator Notes:**	
Not crying, just moaning or snoring noise if anything.<br><br>**Pt will not be on a monitor with BLS only team.**<br>**Still proceed with vital sign changes/behavior changes in scenario, because they should be assessing baby and noting slow RR and noting seizure activity, but will not be on the monitor.**<br><br>-If no intervention to support respirations, can ask Sim engineer to make baby cyanotic.<br>-If still not intervention to support breathing, then just move to State #2 and begin seizing.			**BLS arrives on scene** and begins assessment.<br>-If time is passing and they do not recognize inadequate respirations, you can prompt as Mother- **"It looks like she's not breathing right"**<br>-If no intervention to support respirations, can ask Sim engineer to drop pulse ox to 80s and make baby cyanotic.<br>-If still not intervention to support breathing, then just move to State #2 and begin seizing.	

**State 2**	Actively Seizing		Estimated Time	5min
Vitals		Assessment / Details	Expected Interventions	
**HR**	Abruptly tachycardic to 180	Pupils: PERRL, maybe constricted from prior	* **Expectation #1:** Recognize acute change.<br>* **Expectation #2:** Recognize apnea, inadequate respiratory effort during seizure, start BMV.	
**BP**	120/60	*Lung Sounds: Apneic. Perioral cyanosis*		
**SpO2**	Loss of tracing	*Heart Sounds: Tachycardic*		

<table>
<tr><td>RR</td><td>Apneic</td><td>Seizure: Generalized tonic-clonic seizure.</td><td rowspan="4"></td></tr>
<tr><td>CVP</td><td></td><td rowspan="3">Verbal Response: None.</td></tr>
<tr><td>ETCO2</td><td></td></tr>
<tr><td>OTHER</td><td></td></tr>
<tr><td colspan="3">Notes for Specialist:</td><td>Facilitator Notes:</td></tr>
<tr><td colspan="3">Patient will not be on the monitor with BLS only team.</td><td>Prompt “It looks like the baby isn’t breathing right” or “It looks like the baby is making funny movements, etc” if they do not notice the change.

Have seizure continue until they initiate BVM.</td></tr>
</table>

<table>
<tr><td>State 3</td><td colspan="2">Post-ictal after midazolam administration</td><td>Estimated Time</td><td>(3min)</td></tr>
<tr><td colspan="2">Vitals</td><td>Assessment / Details</td><td colspan="2">Expected Interventions</td></tr>
<tr><td>HR</td><td>130</td><td>Pupils: PERRL</td><td colspan="2" rowspan="7">* Expectation #1: Recognize post-ictal state
* Expectation #2: Recognize risk for hypoventilation while post-ictal

-Will need to support ventilation with either BVM or reposition/OPA and reassess</td></tr>
<tr><td>BP</td><td>90/50</td><td>Lung Sounds: Clear to auscultation *Irregular respiratory effort, snoring sounds</td></tr>
<tr><td>SpO2</td><td>91% if no O2, 98% if on O2</td><td>Heart Sounds: Tachycardic, no murmur, no gallop.</td></tr>
<tr><td>RR</td><td>10</td><td>Seizure: No seizure. Decreased tone (will need to describe)</td></tr>
<tr><td>CVP</td><td></td><td rowspan="3">Verbal Response: Withdraws to pain.</td></tr>
<tr><td>ETCO2</td><td>55</td></tr>
<tr><td>OTHER</td><td></td></tr>
<tr><td colspan="3">Notes for Specialist:</td><td colspan="2">Facilitator Notes:</td></tr>
<tr><td colspan="3">**Depending on how quickly they progress through the scenario, you can have the baby start and stop seizing repeatedly so they can recognize the change, support the airway during seizing, and potentially rotate through different crew members bagging the infant mannequin.**</td><td colspan="2">Prompt to recognize hypoventilation: “It sounds like the baby is snoring.” “Her breathing looks irregular”
Goal is to support hypoventilation.

Depending on how quickly they progress through the scenario, you can have the baby start and stop seizing repeatedly so they can recognize the change, support the airway during seizing, and potentially rotate through different crew members bagging the infant mannequin.

Can end scenario by, “Fast forward a few minutes and you’ve arrived at the ED.”</td></tr>
</table>

	-Have them give report and use that as jumping off point for debrief.

Debrief Points: Infant Seizure **BLS ONLY VERION**
(Tailor to each crew based on what comes up during case)

1) Discuss risk of hypoventilation and promote recognition of hypoventilation in the post-ictal and post-benzodiazepine state

2) BLS- importance of positioning to maintain open airway when seizing and when post-ictal

- Chance to re-iterate importance of positioning to effectively ventilate an infant**
- Depending on comfort level during scenario, may be an opportunity to return to the mannequin and demonstrate good positioning, good seal with mask, and monitoring chest rise for an infant. Can give each person a chance to practice with infant mask if helpful.

3) Monitoring of the post-ictal patient en route is essential

- Use of end-tidal CO2 in post-ictal child, monitoring for inadequate ventilation- Most BLS trucks will not have this, but helpful learning point for when they intercept with ALS.
- *Lots of patients arrive to ED post-ictal, 100% on non-rebreather, but when placed on end tidal are >70!*
- Importance of monitoring vital signs en route to hospital to recognize return of **subtle seizure** activity (eye deviation, tachycardia, hypoventilation)

If you have extra time, can use the opportunity to discuss any piece of infant assessment/management in general. Open to questions or offer tips for examining, assessing, recognizing distress in infants.

**Scenario Title:**	**Simulation 2: Status asthmaticus**<br>**ALS or mixed ALS/BLS VERSION**	**Author:**	
**Department:**	Medicine	**Division:**	Emergency Medicine

**Learning Objectives:**	By the end of this session, participants should be able to:<br>• Objective 1: Recognize pediatric respiratory distress and impending respiratory failure<br>• Objective 2: Recognize presence of criteria for giving IM epi and initiating albuterol for a critically ill asthmatic.<br>• Objective 3: Feel confident administering IM epi to a critically ill asthmatic<br>• Objective 4: Escalate the care of a critically ill asthmatic

**Patient Information:**	**Name**	Charlie Smith	**Age**	5yo
	**Dx**	Asthma	**Gender**	M
	**Sx**	Respiratory distress	**Weight**	18kg (40lbs)
	MRN		**Allergies**	NKDA

**Patient History / Background:**	***"You are first to arrive at the home of a 4yo M w/ history of asthma. Parents called 911 when he developed difficulty breathing. Parent is here and can answer questions. ALS is already en route. Your equipment is here (point to EMS jump bag)."***<br><br>*Additional history if asked:*<br>*-Known asthmatic, uses albuterol but ran out today.*<br>*-Has had cough and congestion for a few days. Much worse today.*<br>*-History of prior ICU admissions for asthma.*<br>*-No fever. No trauma.*<br><br>*PMH: Only asthma*<br>*Meds: Albuterol*<br>*NKDA Immunizations UTD*

**Simulator(s)**		**Monitors**		**Initial Mannequin Props / Set Up:**	
Neonatal		EKG		IV/ART Access:	No access to start
Infant		NIBP			
Pediatric	X	SpO2		Resp Equipment	None to start
Adult		RR			
		ETCO2		Medications	
**To be filled out by SIMPeds**		Temp			
NewBornHAL		ABP		Fluids	
PediHAL		CVP			
Tory		ICP		Props / Moulage	None. Will want mannequin sitting up in tripod posture as best it can.
SimBaby		LAP/RAP			
SimJunior		Others		Equipment needed during simulation	
SimMan		Would you like all monitors to be initially on?<br><br>☐Yes ■o		Epi pen trainer (we will bring)<br>Will use EMS equipment.<br>-Epi pen check and inject kits<br>-Neb mask<br>-IV equipment	
SimMan3G					
SimManEssential					

			-Meds for mag, solumedrol, NS

**State 1**	Severe respiratory distress		Estimated Time	(3min)
Vitals		Assessment / Details	Expected Interventions	
**HR**	140	Pupils: PERRL	***Expectation #1:** Recognize critical illness, activate ALS<br><br>***Expectation #2:** Recognize severe asthma exacerbation with respiratory distress.<br><br>**Expectation #3:** Initiate O2<br><br>**Expectation #4:** Initiate albuterol<br><br>**Expectation #5:** Consider IM epinephrine	
**BP**	80/40	*Lung Sounds: Inspiratory and expiratory wheezing bilaterally*		
**SpO2**	88%	*Heart Sounds: Tachycardic, no murmur, no gallop*		
**RR**	50-60	*Seizure: No.*		
**CVP**		*Verbal Response: Moaning or gasping. "I (pause) can't (pause) breathe"*<br>*Not able to speak sentences due to respiratory distress.*<br><br>****Should NOT sound anything like stridor***		
**ETCO2**				
**OTHER**				
Notes for Specialist:			**Facilitator Notes:**	
Monitor is off until ALS arrives and places leads.<br>Starts off awake, but gasping when talking. "I (pause) can't (pause) breath"<br><br>*Will move quickly to next stage- just moaning in response to questions.*			Prompt to recognize tachypnea and wheezing if they don't.	
**State 2**	Impending respiratory failure		Estimated Time	(4min)
Vitals		Assessment / Details	Expected Interventions	
**HR**	140	Pupils: PERRL	* **Expectation #1:** Recognize impending respiratory failure.<br>* **Expectation #2:** Administer IM epinephrine for impending respiratory failure.<br>* **Expectation #3:** Administer albuterol for severe asthma.<br><br>**Criteria for BLS Use of IM epi:**<br>• >6mo<br>• Hx of asthma or home albuterol<br>• Resp arrest or approaching arrest<br>• Diminished or absent breath sounds<br>**BLS IM epi use:**<br><25kg: 0.15mg<br>>25kg: 0.3mg<br>*Contact medical control for second dose if needed at 5min.	
**BP**	80/40	*Lung Sounds:* ***No aeration bilaterally****, still very tachypneic*		
**SpO2**	89%	*Heart Sounds: Tachycardic, no murmur, no gallop*		
**RR**	50-60	*Seizure: No*		
**CVP**		*Verbal Response: Now w/ poor respiratory effort. Lethargic.*<br><br>*Just moans- doesn't speak or answer questions.*		
**ETCO2**	34 (shark fin shaped tracing)			
**OTHER**	Lethargic, minimally responsive			
Notes for Specialist:			**Facilitator Notes:**	

Just moans- doesn't speak or answer questions.<br><br>*If IM epi is not given, remain in stage 2 indefinitely.*	**Prompt to recognize lethargy/somnolence:** "Why isn't he talking anymore?" "Why is he so **lethargic?"**<br><br>-Can prompt to re-listen, comment on poor aeration.<br><br>Have this stage of ***impending* respiratory failure** last as long as possible for them to get **IM epi** in without pushing it towards intubation.

**State 3**	Improvement after epi		Estimated Time	(5min)
Vitals		Assessment / Details	Expected Interventions	
**HR**	150	Pupils: PERRL	Prompt for ALS arrival at this point- bedside report from BLS<br><br>* **Expectation #1:** Recognize improvement after IM epi.<br>* **Expectation #2:** Manage severe asthma:<br>-Non-rebreather<br>-Albuterol<br>-Magnesium<br>-Solumedrol<br>-NS bolus	
**BP**	96/50	*Lung Sounds: Wheezing throughout, improved air movement*		
**SpO2**	92%	*Heart Sounds: Tachycardic, no murmur*		
**RR**	36	*Seizure: None*		
**CVP**	34 (shark fin shaped tracing)	*Verbal Response: Short sentences. Still breathless.*		
**ETCO2**				
**OTHER**				

Notes for Specialist:	**Facilitator Notes:**
Can just moan to IM epi administration<br>*After IM epi is given, patient starts to verbalize more, "I can breathe"<br><br>**Still in distress- short, gaspy response to questions:**<br>"A little better."<br>"Still (breath) hard (breath) to breath."<br>Can moan, "Ouch- that hurts" to IV access.	Goal will be to recognize improvement after IM epi (don't want to arrest, don't want to intubate), but still is sick, hypoxic, wheezing, tachypneic. Still needs albuterol/ipratropium, Mag, solumedrol.<br><br>Can end scenario by, "Fast forward a few minutes and you've arrived at the ED." -Have them give report and use that as jumping off point for debrief.

<u>Debriefing Points: Severe Asthma- ALS or mixed ALS/BLS VERSION</u>
(Tailor to each crew depending on what comes up during case)

1) Recognition of severe asthma- Can discuss visibility of accessory muscle use in children, past admissions and ICU as indicators of severe disease

2) Indications for IM epi in asthma

   *This is a new protocol for BLS. Some teams may not be aware that they are allowed to do this. They definitely are! A great time to educate on this new tool in the toolbox of BLS care for a critically ill child.

   Criteria for BLS Use of IM epi:
   - >6mo
   - Hx of asthma or RAD or home albuterol
   - Resp arrest or approaching resp arrest
   - Diminished or absent breath sounds

   BLS IM epi use:
   <25kg: 0.15mg
   >25kg: 0.3mg
   *Contact medical control for second dose if needed at 5min.

3) ALS management of severe asthma- Role of albuterol, steroids, magnesium (weight based dosing)
4) If asked, can discuss the challenges of intubation and ventilation of asthmatics, but the goal is to avoid intubating asthmatics and manage medically and via non-invasive given challenges
5) If you go through this fast, also an opportunity to talk about any pediatric assessment pearls, communicating with worried parents, etc.

**Scenario Title:**	**Simulation 2: Status asthmaticus**<br>****BLS ONLY VERSION****	**Author:**	
**Department:**	Medicine	**Division:**	Emergency Medicine

Learning Objectives:

By the end of this session, participants should be able to:

- Objective 1: Recognize pediatric respiratory distress and impending respiratory failure
- Objective 2: Recognize presence of criteria for giving IM epi and initiating albuterol for a critically ill asthmatic.
- Objective 3: Feel confident administering IM epi to a critically ill asthmatic

Patient Information:

**Name**	Charlie Smith	**Age**	5yo
**Dx**	Asthma	**Gender**	M
**Sx**	Respiratory distress	**Weight**	18kg (40lbs)
MRN		**Allergies**	NKDA

Patient History / Background:

"You are first to arrive at the home of a 4yo M w/ history of asthma. Parents called 911 when he developed difficulty breathing. Parent is here and can answer questions. ALS is already en route. Your equipment is here (point to EMS jump bag)."

<u>Additional history if asked:</u>
-Known asthmatic, uses albuterol but ran out today.
-Has had cough and congestion for a few days. Much worse today.
-History of prior ICU admissions for asthma.
-No fever. No trauma.

PMH: Only asthma
Meds: Albuterol
NKDA Immunizations UTD

**Simulator(s)**		**Monitors**		**Initial Mannequin Props / Set Up:**	
Neonatal		EKG		IV/ART Access:	No access to start
Infant		NIBP			
Pediatric	X	SpO2		Resp Equipment	None to start
Adult		RR			
		ETCO2		Medications	
**To be filled out by SIMPeds**		Temp			
NewBornHAL		ABP		Fluids	
PediHAL		CVP			
Tory		ICP		Props / Moulage	None. Will want mannequin sitting up in tripod posture as best it can.
SimBaby		LAP/RAP			
SimJunior		Others		Equipment needed during simulation	
SimMan		Would you like all monitors to be initially on?<br>☐Yes ∎o		Epi pen trainer (we will bring)<br><u>Will use EMS equipment.</u><br>-Epi pen check and inject kits<br>-Neb mask	
SimMan3G					
SimManEssential					

**State 1**	Severe respiratory distress		Estimated Time	(3min)
Vitals		Assessment / Details	Expected Interventions	
**HR**	140	Pupils: PERRL	***Expectation #1:** Recognize critical illness<br><br>***Expectation #2:** Recognize severe asthma exacerbation with respiratory distress.<br><br>**Expectation #3:** Initiate O2<br><br>**Expectation #4:** Initiate albuterol<br><br>**Expectation #5:** Consider IM epinephrine	
**BP**	80/40	*Lung Sounds: Inspiratory and expiratory wheezing bilaterally*		
**SpO2**	88%	*Heart Sounds: Tachycardic, no murmur, no gallop*		
**RR**	50-60	*Seizure: No.*		
**CVP**		*Verbal Response: Moaning or gasping. "I (pause) can't (pause) breathe"*<br>*Not able to speak sentences due to respiratory distress.*<br><br>****Should NOT sound anything like stridor***		
**ETCO2**				
**OTHER**				
Notes for Specialist:			**Facilitator Notes:**	
**Will not be on monitor for BLS-only cases**<br><br>Starts off awake, but gasping when talking. "I (pause) can't (pause) breath"<br><br>*Will move quickly to next stage- just moaning in response to questions.*			Prompt to recognize tachypnea and wheezing if they don't.	

**State 2**	Impending respiratory failure		Estimated Time	(4min)
Vitals		Assessment / Details	Expected Interventions	
**HR**	140	Pupils: PERRL	* **Expectation #1:** Recognize impending respiratory failure.<br>* **Expectation #2:** Administer IM epinephrine for impending respiratory failure.<br>* **Expectation #3:** Administer albuterol for severe asthma.<br><br>**Criteria for BLS Use of IM epi:**<br>• >6mo<br>• Hx of asthma or home albuterol<br>• Resp arrest or approaching arrest<br>• Diminished or absent breath sounds<br>**BLS IM epi use:**<br><25kg: 0.15mg<br>>25kg: 0.3mg<br>*Contact medical control for second dose if needed at 5min.	
**BP**	80/40	*Lung Sounds:* ***No aeration bilaterally****, still very tachypneic*		
**SpO2**	89%	*Heart Sounds: Tachycardic, no murmur, no gallop*		
**RR**	50-60	*Seizure: No*		
**CVP**		*Verbal Response: Now w/ poor respiratory effort. Lethargic.*<br><br>*Just moans- doesn't speak or answer questions.*		
**ETCO2**	34 (shark fin shaped tracing)			
**OTHER**	Lethargic, minimally responsive			
Notes for Specialist:			**Facilitator Notes:**	

Just moans- doesn't speak or answer questions.<br><br>*If IM epi is not given, remain in stage 2 indefinitely.*	**Prompt to recognize lethargy/somnolence:** "Why isn't he talking anymore?" "Why is he so **lethargic?"**<br><br>-Can prompt to re-listen, comment on poor aeration.<br><br>Have this stage of ***impending* respiratory failure** last as long as possible for them to get **IM epi**. If they are not getting there, prompt by parent "Last time he was this sick they gave him the shot in the leg"

**State 3**	Improvement after epi		Estimated Time	(5min)
Vitals		Assessment / Details	Expected Interventions	
**HR**	150	Pupils: PERRL	***Expectation #1:** Recognize improvement after IM epi.<br>* **Expectation #2:** Manage severe asthma:<br>-Albuterol neb<br>-Rapid transport	
**BP**	96/50	*Lung Sounds: Wheezing throughout, improved air movement*		
**SpO2**	92%	*Heart Sounds: Tachycardic, no murmur*		
**RR**	36	*Seizure: None*		
**CVP**	34 (shark fin shaped tracing)	*Verbal Response: Short sentences. Still breathless.*		
**ETCO2**				
**OTHER**				

Notes for Specialist:	**Facilitator Notes:**
Can just moan to IM epi administration<br>*After IM epi is given, patient starts to verbalize more, "I can breathe"<br><br>**Still in distress- short, gaspy response to questions:**<br>"A little better."<br>"Still (breath) hard (breath) to breath."	Goal will be to recognize improvement after IM epi (don't want to arrest), but still is sick, hypoxic, wheezing, tachypneic. Still needs albuterol.<br><br>Can end scenario by, "Fast forward a few minutes and you've arrived at the ED." -Have them give report and use that as jumping off point for debrief.

Debriefing Points: Severe Asthma- BLS ONLY VERSION
(Tailor to each crew depending on what comes up during case)

1) Recognition of severe asthma- Can discuss visibility of accessory muscle use in children, past admissions and ICU as indicators of severe disease.

2) Indications for IM epi in asthma

   *This is a new protocol for BLS. Some teams may not be aware that they are allowed to do this. They definitely are! A great time to educate on this new tool in the toolbox of BLS care for a critically ill child.

   Criteria for BLS Use of IM epi:
   - >6mo
   - Hx of asthma or RAD or home albuterol
   - Resp arrest or approaching resp arrest
   - Diminished or absent breath sounds

   BLS IM epi use:
   <25kg: 0.15mg
   >25kg: 0.3mg
   *Contact medical control for second dose if needed at 5min.

3) If you go through this fast, also an opportunity to talk about any pediatric assessment pearls, communicating with worried parents, etc.

Newborn Resuscitation

*If BLS-only crew, will just do Scenario #1/Baby #1. If mixed BLS/ALS crew, can run Scenario #1/Baby #1 for initial 2-3 responders (BLS if they have not already participate hands on today), then have Baby #2 deliver and ALS crew arrive on scene**.**

***Quick intro to manikin before starting the scenario with Sim engineer- The newborn mannequins are more fragile than the others.**

-**Baby A** will be on **stretcher** and play to roll off to side for 2nd crew to care for **Baby B** on the adjacent **table**.

**Scenario Title:**	Newborn resuscitation		**Author:**	Adapted from Texas Children's Hospital Pedi-STEPPS
**Department:**	Pediatrics	**Division:**	Emergency Medicine	
**Learning Objectives:**	By the end of this session, participants should be able to:<br>• Demonstrate basic skills of neonatal resuscitation per EMS protocol<br>• Review possible etiologies of distress in the newborn<br>• Identify particular vulnerabilities of the newborn<br>• Review key features of history when assessing a newborn			

**Patient Information:**	**Name**	Baby Davis	**Age**	0d
	**Dx**	Newborn	**Gender**	
	**Sx**	Apnea	**Weight**	3kg
	MRN		**Allergies**	NKDA

**Patient History / Background:**	**"You are called to the home of a pregnant woman in labor. You arrive to find she has just delivered. Mother's water broke this morning, family arrived to take her to hospital, but she delivered precipitously.**<br><br>**There is another EMS crew on scene attending to Mother. Your EMS crew is next to arrive on scene.**<br>**Your equipment is here- (point to bag). Family members are here and can answer questions you have."**<br><br>*If asked: Mother is 30yo, otherwise healthy. 35wk twin pregnancy, no complications.*<br>*-Routine prenatal care.*<br>*-Normal anatomy scans and routine ultrasounds.*<br>*-This is her 4th pregnancy.*

**Simulator(s)**		**Monitors**		**Initial Mannequin Props / Set Up:**	
Neonatal	X	EKG		IV/ART Access:	No access to start. No monitors on to start (will need EKG, BP, SpO2, and RR once ALS arrives and places patient on monitor)
Infant		NIBP			
Pediatric		SpO2		Resp Equipment	
Adult		RR			
		ETCO2		Medications	
**To be filled out by SIMPeds**		Temp			
NewBornHAL	X	ABP		Fluids	
PediHAL		CVP			

Tory		ICP		Props / Moulage	Umbilical stump present, **clamp on.** No umbilical lines in place.<br>Amniotic fluid moulage
SimBaby		LAP/RAP			
SimJunior		Others		Equipment needed during simulation	
SimMan		Would you like all monitors to be initially on?<br>**No monitors initially**<br>☐Yes ☐No		-Newborn/infant bag-valve-mask<br>-Newborn/infant non-rebreather<br>-Newborn/pediatric OPA and NPA<br>-Supplemental oxygen and tubing<br>-IO<br>-Saline flushes<br>-Syringes for epinephrine<br>-Blankets<br>-Umbilical cord clamps/Kelly clamps<br>-Trauma shears<br>-Bulb suction x2	
SimMan3G					
SimManEssential					

**State 1**	Newly born- BLS EMS crew arrives	Estimated Time	(1-2min)
Vitals		Assessment / Details	Expected Interventions
**HR**	50	Pupils: Eyes closed, pupils equal and reactive if asked	*** Expectation #1:**<br>-Rapidly assess the newly born patient<br><br>**Expectation #2:**<br>-Dry, suction, and stimulate<br><br>* **Expectation #3**<br>Recognize poor tone, no/minimal respiratory effort<br>-Recognize presence of pulse
**BP**	45/24	Lung Sounds: Apnea. Can have occasional, irregular grunting breaths at ~5x per minute.	
**SpO2**	n/a	Heart Sounds:RRR, no murmur<br>Pulse palpable at umbilical stump if possible	
**RR**	0-5	Seizure: No seizure activity. Limp/low tone if possible.	
**CVP**		Verbal Response: No crying.<br><br>If asked: skin color is cool and **cyanotic**	
**ETCO2**			
**OTHER**	Temp 95.8 if asked		

Notes for Specialist:	**Facilitator Notes:**
**Pulse palpable at umbilical stump**<br>**Cyanotic-** set pulse ox for perioral cyanosis, facilitator will announce central cyanosis<br>**Limp/low tone** if possible, if not, then just no movement.	*If asked: Mother is healthy 30yo. 35wk twin pregnancy, no complications, 4th pregnancy.*<br>*-Routine prenatal care.*<br>*-Normal anatomy scans and routine ultrasounds.*<br><br>**Exam findings to announce:**<br>-Central cyanosis<br>-Limp/low tone<br>-Pulse palpable at umbilical stump

**State 2**	Still Apneic		Estimated Time	(60sec)
Vitals		Assessment / Details	Expected Interventions	
**HR**	40-60	Pupils: Eyes closed, pupils equal and reactive if asked	*** Expectation #1**<br>-Recognize ongoing apnea despite dry/bulb suction/stimulate<br>-Reposition/suction airway<br>-Ventilate via Bag valve mask<br><br>*** Expectation #2**<br>-Consider access, consider ALS intercept if BLS only crew so far	
**BP**	40/20	Lung Sounds: No spontaneous respiratory effort. Lung sounds clear when bagging. Chest rise symmetric when bagging.		
**SpO2**	N/A	Heart Sounds: Normal. No murmur, no gallop. Pulse present at umbilical stump if possible.		
**RR**	0-5	Seizure: No seizure activity. Limp/low tone if able.		
**CVP**		Verbal Response: **No crying**.<br><br>Skin remains cool, **cyanotic**		
**ETCO2**				
**OTHER**				
Notes for Specialist:			**Facilitator Notes:**	
**Remains limp, cyanotic, no crying**			If time is passing and they do not recognize inadequate respirations, you can prompt as Mother/family- **"It looks like he's not breathing! Why isn't he crying?! Why is he blue/dusky?"**	

**State 3**	Responding to positive pressure		Estimated Time	(60sec)
Vitals		Assessment / Details	Expected Interventions	
**HR**	~140 (Rapidly responds from 60 to >100 after BVM)	Pupils: Eyes closed, PERRL if asked	*** Expectation #1:**<br>-Administers BVM ventilation at **40-60 bpm**<br>-Administers BVM for at least 60sec before chest compressions<br><br>*** Expectation #2**<br>-Recognizes response to BVM ventilation (improved HR, spontaneous respirations to start, then starts to cry)<br><br>**Verbalize routine care en route:**<br>-Keep warm<br>-Closely monitor during transport<br>-Gather additional history from family	
**BP**	60/30	Lung Sounds: Clear to auscultation. Symmetric, regular chest rise		
**SpO2**	If pulse ox applied, now 98%	Heart Sounds: **HR improves with 20-30sec** of BVM. RRR, no murmur, no gallop. **Cyanosis resolves with 30sec of BVM.**		
**RR**	36	Seizure: No seizure. Improved tone. Normal movements of extremities if able.		
**CVP**		Verbal Response: After BVM for 30-60sec, baby **starts to cry.**		
**ETCO2**				
**OTHER**				
Notes for Specialist:			**Facilitator Notes:**	
Within 20-30sec of BVM ventilation, baby can begin spontaneously breathing.<br>HR should climb quickly with the start of BVM ventilation<br>After 1min of ventilation, baby can start with weak cry.<br>Stronger cry at end of scenario.			If this takes a long time, end scenario by, "Fast forward to your arrival at the ED."<br>-Have them give report and use that as jumping off point for debrief.	

	Can discuss newborn vulnerabilities and routine newborn care in debrief<br><br>Allow them to enjoy the crying infant for 20sec, then "transport" first baby and **Baby #2 is delivered move to next case below.**

<u>*If BLS only crews, stop here. Or deliver Baby 2 to allow 2 more people to participate, but stop after initiation of chest compressions with baby responding, breathing, and crying.</u>

<u>Baby #2</u>

**State 1**	Baby #2 Born, 2nd EMS crew/ALS team arrives	
	**Patient History / Background:**	**"Mother (being cared for by another EMS crew) has now delivered the second infant. The baby is not moving and not breathing."<br>*If the initial crew called for ALS, announce, "Your ALS crew has arrived."<br>*If they didn't call for ALS, announce, "An additional ALS crew was automatically dispatched to this call and has now arrived"**

Vitals		Assessment / Details	Expected Interventions
**HR**	40-60	Pupils: Eyes closed, pupils equal and reactive if asked	*** Expectation #1**<br>-Dry, suction, stimulate<br>-Recognize ongoing apnea despite dry/suction/stimulate<br>-Reposition/suction airway<br>-Ventilate via Bag Valve Mask
**BP**	40/20	Lung Sounds: No spontaneous respiratory effort. Lung sounds clear when bagging. Chest rise symmetric when bagging.	
**SpO2**	N/A	Heart Sounds: Normal. No murmur, no gallop. Pulse present at umbilical stump if possible.	
**RR**	0-5	Seizure: No seizure activity. Limp/low tone if able.	
**CVP**		Verbal Response: No crying.<br><br>Skin remains cool, **cyanotic**	
**ETCO2**			
**OTHER**			

Notes for Specialist:	**Facilitator Notes:**
**Pulse palpable at umbilical stump**<br>**Cyanotic-** set pulse ox for perioral cyanosis, facilitator will announce central cyanosis<br>**Limp/low tone** if possible, if not, then just no movement.	Allow the newly arriving crew to assess Ba<br>-Same prompts as above if not recognizin<br>apnea/bradycardia.<br>"Why isn't she crying? Why is she blue? Is she breathing?"

**State 2**	Decompensation/No response to BVM		Estimated Time	2min
Vitals		Assessment / Details	Expected Interventions	
**HR**	40	Pupils: Eyes closed, PERRL if asked	*** Expectation #1:**<br>-Recognize still apneic and bradycardic after 60sec of BVM<br>-Initiate chest compressions	
**BP**	30/20	Lung Sounds: No grunting		
**SpO2**	No pleth	Heart Sounds: Bradycardic		
**RR**	Apneic			
**CVP**		Verbal Response: **No crying**. Limp/low tone if able.<br>**Cyanotic** if able/if asked.		
**ETCO2**				
**OTHER**				
Notes for Specialist:			**Facilitator Notes:**	

**Limp, cyanotic, not crying.**			
**State 3**	No change	Estimated Time	3-4min
Vitals		Assessment / Details	Expected Interventions
**HR**	40	Pupils: Eyes closed, PERRL if asked	* **Expectation #1:** Obtain IV/IO access<br><br>* **Expectation #2:** Administer NS (10mL/kg [30mL])<br><br>***Expectation #3:** Consider intubation (OK if they don't as long as they're getting good chest rise with BVM)<br><br>**Expectation #4:** Discuss medical control for epinephrine order<br>-If requested, administer Epinephrine (0.01mg/kg 1:10,000 or (0.1mg/ml=0.03mg) (EMS protocols still use 1:10,000 notation)
**BP**	30/20	Lung Sounds: Bilateral breath sounds with ventilation	
**SpO2**	Poor tracing/No signal	Heart Sounds: **Bradycardic**	
**RR**	(rate they are bagging)		
**CVP**		Verbal Response: **No crying**	
**ETCO2**	34 (only if intubated)		
**OTHER**			
Notes for Specialist:			**Facilitator Notes:**
**Cyanotic** if able.<br>Limp/poor tone if able.<br>**No crying**.			If no IV/IO, no fluids, no call to medical control, you can let them linger with the baby in extremis for a minute or two.<br>**Then prompt them:**<br>"Is there anything else you could do the support the circulation at this point?"<br>"Are there potential interventions that require medical control input?"<br>If they don't place IO/intubate/or call Medical Control for epi, don't leave them in endless CPR.<br>**Let baby respond and plan to discuss next steps in the code during debrief.**<br>If they don't know how to access real-time medical control, prompt them to call CMED to connect them with hospital.
**State 4**	Improvement	Estimated Time	1-2min
Vitals		Assessment / Details	Expected Interventions
**HR**	120-130	Pupils: PERRL	* **Expectation #1:** Warm infant<br>* **Expectation #3:** Prepare for transport<br>-Warm up ambulance, plan for securing Pt to stretcher<br>* **Expectation #4:** Score APGAR
**BP**	50/35	Lung Sounds: Bilateral breath sounds with BVETT	
**SpO2**	95%	Heart Sounds: No gallop	
**RR**	(at rate they are bagging)		
**CVP**		Verbal Response: Can start to cry.	
**ETCO2**	40		
**OTHER**	If asked 97F		
Notes for Specialist:			**Facilitator Notes:**
**Improved color, resolved cyanosis.**<br>**Improved tone, spontaneous movement**			End scenario, "Fast forward to arrival at ED."

	-Have them give report and use that as jumping off point for debrief.<br>*Can discuss newborn vulnerabilities and routine newborn care in debrief*

MA Statewide Newborn Protocols

Routine EMS Newborn Assessment:

-No routine suctioning (even with meconium) if infant is crying or no respiratory distress
-Clamp and cut cord (leave 6 inches) after initial assessment and cord stops pulsating

-If asked about delayed cord clamping, current ACOG recommendation for delayed cord clamping of 30-60sec for benefit of infant, no need for longer delay.

-Rapidly dry and warm, **remove wet blankets**, cover newborn's head
-If respiratory distress or HR<100, proceed to Newborn Resuscitation (below)
-Apgar scores at 1 and 5min* (discussion below)
-Special notation in EMS protocol that newborns are prone to hypothermia which increases risk of hypoglycemia, hypoxia, lethargy. Encourages: Drying, swaddling, warm blankets, and raising temperature inside ambulance.
-Special notation of central cyanosis vs peripheral as peripheral cyanosis is normal in newborns
*Skin to skin is not specifically mentioned in their protocol (I think reasonable to do in the stable infant/stable mother at home, but not a safe mode of transportation for the infant).

EMS Newborn Resuscitation:

-Open and suction airway. Suction hypopharynx if meconium present only if **non-vigorous**
-Ventilate at 40-60 breaths per minute

****This is going to feel very fast to them. Discuss rate, allowing exhalation, and being careful to bag only to chest rise and not let adrenaline drive big volumes/high pressures.**

-If HR <60, ventilate x1min
-If HR<60 after 1min of ventilation, initiate chest compressions

ALS standing orders:

-If meconium present and infant not vigorous, consider ETI and suctioning
-If HR 60-80 and rapidly rising, continue manual ventilation at 40-60 bpm, monitor for dysrhythmias
-If HR< 60 despite ventilation x1min, initiate chest compressions, advanced airway management (with capnography)
-If defibrillation is needed: 2J/kg initial, 4J/kg subsequent
-If synchronized cardioversion is needed, 0.5-1J/kg
-Establish IV/IO access, treat for shock with 10cc/kg NS over 5-10min
-Medical control may order: 0.01-0.03mg/kg epinephrine 1:10,000 (0.1 mg/ml), IV/IO

Debriefing Points:

(Tailor to each crew based on what comes up during case)

*Acknowledge that this might be a once in a career call for most crews. Some areas have lots of unplanned home deliveries. Other areas this is really rare.

1. **Appropriate routine care of the well newborn** (review the steps above)

2. Understanding of **basic skills of neonatal resuscitation** per EMS protocol (can review the steps as above)

   ***Most** term or near-term infants will respond to warm/dry/stimulate and bag-mask ventilation.

-The most important piece of newborn resuscitation is an open airway, good positioning, and BVM ventilation.
-This will be an adrenaline-filled situation and they will be bagging at higher rates than usual- discuss risk of barotrauma and the importance of bagging only to chest rise and not using high pressures.
-Can discuss neonatal intubation and choosing tube size based on gestational age, but reiterate importance of good seal, BVM with adequate rate, and good (not aggressive) chest rise.

3. Understanding of the differential diagnosis for the newborn in distress
   -**Newborn is vulnerable to**:
      -Hypovolemia if Mother has been hemorrhaging
      -Sepsis if Mother is infected/febrile
      -Hypothermia if not quickly dried/wrapped
      -Hypoglycemia if pre-term or SGA or hypothermic or Mother with gestational DM
      -Undiagnosed congenital anomalies if no prenatal care

4. **Important pieces of maternal history for newborn care**:
      -Term or preterm? Premature infants are at higher risk for:
         -Needing respiratory support due to immature lung development and immature respiratory drive
         -Hypothermia (less body fat)
         -Hypoglycemia
      -Routine prenatal care? Routine ultrasounds? Any known fetal anomalies?
      -Maternal history of infection during pregnancy or fever during delivery?
      -Maternal medication use
         -Diabetic mothers are more likely to have infants with hypoglycemia
         -Mothers using opioids are more likely to have infants with respiratory issues

APGAR Scale

Feature Evaluated	2 Points	1 Point	0 Points
**A**ctivity (Muscle Tone)	Active Movement	Arms and legs flexed (Weak, some movement)	Limp or flaccid
**P**ulse	Over 100 bpm	Below 100 bpm	Absent
**G**rimace (Irritability/reflexes)	Cry, sneeze, cough, active movement	Grimace (some flexion of extremities)	No reflexes
**A**ppearance (Skin Color)	Completely pink	Body pink, Extremities blue	Blue, pale
**R**espiration	Vigorous cry Full breaths	Slow, irregular, or gasping breaths, weak cry	Absent

*Acknowledge that calculating the Apgar score is a low priority- if a newborn has a poor Apgar score the 2 EMTs caring for the baby will be too busy actively trying to address the problem to pause and calculate Apgar.
-It is helpful, once the dust settles and the baby is stabilized, to retrospectively consider what the score was.
-This is objective information for the receiving hospital to understand how "down" the baby was and how long.
-Useful if a potential candidate for cooling.
-Helps hospital understand the work the EMS crew did to bring a baby from poor initial Apgar to vigorous baby.

<u>Endotracheal tube sizing for newborns:</u>
Gestational age=tube size for uncuffed
25wk=2.5
30wk=3.0
35wk=3.5
Term- still likely 3.5
<u>Consider using cuffed tubes</u> for neonates if available for stability in transport. This may not be widely available as historically only uncuffed were used.
Also note, **neonatal LMAs and supraglottic airways** are now available for EMS and are a great option if they have having difficulty bag-valve masking.

*If you have extra time, can use the opportunity to discuss any piece of newborn/infant assessment/management in general. Open to questions or offer tips for examining, assessing, recognizing distress in newborns/infants.

Weight Based Medication Dosing per EMS Protocols

- Albuterol
    - <2yo: 1.25mg in 3mL normal saline nebulized, X1 dose
    - >2yo 2.5-3mg in 3mL normal saline nebulized, x1 dose
    - Paramedics may also administer ipratropium
- Epinephrine
    - Newborn resuscitation: 0.01-0.03mg/kg of 1:10,000 (0.1mg/ml) IV/IO
        - Infusion after resuscitation: 0.1-1mcg/kg/min
    - Bronchospasm with severe distress
        - >6mo and <25kg: 0.15mg IM (autoinjector)
        - >25kg: 0.3mg IM (autoinjector)
- Glucose/Dextrose
    - Known Hypoglycemia (glucose <70mg/dL)
        - Dextrose 10% 0.5gm/kg IV/IO
        - Glucagon 0.1 mg/kg IV/IO/IM/IN/SC (max 1mg)
    - Known Hyperglycemia
        - Administer 20ml/kg fluid bolus
- Hydrocortisone
    - 2mg/kg IV/IO/IM (max 100mg)
- Magnesium
    - 25mg/kg IV/IO over 10min (max 2g)
- Methylprednisone
    - 2mg/kg IV/IO/IM (max 125mg)
- Midazolam
    - 0.05mg/kg IV/IO/IM (max 4mg single dose)
    - 0.2mg/kg IN (max 10mg)
- Racemic epi
    - 11.25mg in 2.5ml normal saline for severe croup with stridor at rest

EMS RSI Waiver: (This protocol has been changed since to include rocuronium, but this list as per 2019 curriulum)
(Limited to few cities with OEMS waiver):
Fentanyl 1-2 mcg/kg and / or versed 0.05-0.1 mg/kg
Atropine 0.01mg/kg IV (Pt less than 30 kg)
Etomidate 0.2-0.3 mg/kg MAX 20mg - only in children >10 yr old
Succinylcholine 1.5-2.0 mg/kg

Print and attach MA EMS Protocol PDFs to Instructor Guide

- Seizure
- Asthma
- Newborn Care
- Newborn Resuscitation
- Trach care
- Weight based pediatric medications

EMSC Simulation & Education Day

Assessment

1. Which position best represents the optimal position to open the airway?

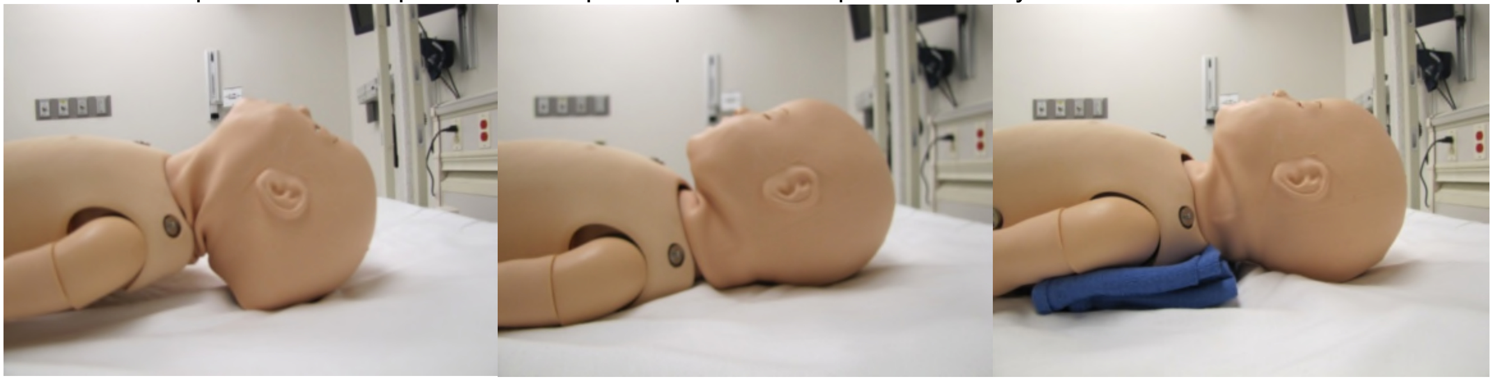

A. B. C.

2. What is the most important initial action to perform in an unresponsive newborn?
    a. Provide oxygen via simple face mask
    b. Perform chest compressions
    c. Ventilate the lungs with a bag valve mask (BVM)
    d. Give epinephrine

3. Pediatric patients with ineffective ventilation require repositioning and bag valve mask (BVM) ventilation to prevent progression to respiratory arrest. Which of the following patients has signs of ineffective ventilation or impending respiratory failure?
    a. A newborn breathing 40 times per minute
    b. An 8 month old with bronchiolitis with occasional retractions
    c. An 18 month old with irregular snoring respirations after a febrile seizure
    d. A 15 year old who is intoxicated but speaking in sentences

4. You are called to the home of a 3 year old male with a history of asthma. Parents report they ran out of his albuterol overnight. He is lethargic with poor respiratory effort. There are diminished breath sounds bilaterally. You estimate his weight at 15kg. Your partner provides bag valve mask (BVM) ventilations and you give:
    a. Epinephrine 0.15mg (Epi-Pen Junior) IM
    b. Epinephrine 0.3mg (Epi-Pen) IM
    c. Epinephrine 0.15mg IV
    d. No medications

5. American Heart Association guidelines for 2 rescuer infant CPR include:
    a. Compression/ventilation ratio 30:2 and compression depth of at least 1/3 of the chest
    b. Compression/ventilation ratio 30:2 and compression depth of at least 1/2 of the chest
    c. Compression/ventilation ratio 15:2 and compression depth of at least 1/3 of the chest
    d. Compression/ventilation ratio 15:2 and compression depth of at least 1/2 of the chest

6. You arrive on the scene of an actively seizing 10 year old patient. He is pink and well perfused, but has loud, snoring respirations at a rate of 18 breaths per minute. The most important initial step is to:
    a. Reposition the airway using a chin-lift or jaw-thrust maneuver
    b. Call ALS for intubation
    c. Start bag valve mask (BVM) ventilation
    d. Place the patient prone to open the airway

7. Which of the following can cause <u>difficulty</u> bag valve mask (BVM) ventilating an infant:
    a. Slow delivery of each breath over 1 second
    b. Hyperextension of the neck
    c. Applying a shoulder roll and extension of the head
    d. A mask size that extends from the bridge of the nose to the cleft of the chin

8. An oral and nasal airway should be:
   a. Cleaned well for reuse after the transport
   b. Inserted in all critically injured patients
   c. Used to keep the tongue from blocking the airway
   d. Used in order to prevent the need for suctioning

9. Normal vital signs for a 7 month old infant are:

a.	BP: 130/72	RR: 20/min	P: 180/min	SpO_2: 99%
b.	BP: 90/50	RR: 22/min	P: 130/min	SpO_2: 99%
c.	BP: 96/54	RR: 44/min	P: 185/min	SpO_2: 97%
d.	BP: 120/80	RR: 16/min	P: 70/min	SpO_2: 99%

10. A 7 year old male is having difficulty breathing after playing in the schoolyard. He is alert, frightened, breathing 24 times per minute, and has a pulse rate of 130 beats per minute. You note wheezing in all lung fields. His skin is warm and diaphoretic. You should:
   a. Assist with the administration of the patient's multi-dose albuterol inhaler
   b. Assist with the patient's prescribed epinephrine auto-injector
   c. Assist the patient's breathing with a bag valve mask (BVM)
   d. Transport immediately and notify the parents while enroute

11. You are the first responder to the home for a sudden vaginal delivery of a full term newborn infant. Your first treatment should be:
   a. Apply oxygen
   b. Bag valve mask (BVM) ventilate
   c. Suction, dry, and stimulate
   d. Assess blood pressure and glucose

12. Newborns who are born prematurely are at a higher risk for all of the following **except**:
   a. Inadequate respiratory effort
   b. Hypoglycemia
   c. Hypothermia
   d. Supraventricular tachycardia

13. You are performing rescue breaths using a bag valve mask (BVM) attached to 100% O_2. After several breaths you notice the patient's O_2 saturation has dropped significantly. What should you do next?
   a. Increase the rate of respirations
   b. Reposition the patient's head
   c. Change the seal on the mask
   d. Remove the oral airway from the patient's mouth

14. All of the following can cause poor or inadequate ventilation in a post-ictal child **except**:
   a. Suppression of the respiratory drive by antiepileptic medications
   b. Loss of muscle tone in the soft tissues of the airway and pharynx
   c. Hyperglycemia due to stress response
   d. Recurrence of seizure activity

15. When performing 2-person CPR on a 4 year old child, the proper compression to ventilation ratio is:

a. 10:2
b. 15:2
c. 20:2
d. 30:2

16. The normal respiratory rate for a healthy, full-term newborn is:

a. 12/minute
b. 24/minute
c. 48/minute
d. 72/minute

17. You are called for the imminent delivery of a full-term male infant. The baby is limp and not breathing spontaneously. You dry and stimulate the baby. The heart rate is 40bpm. Your next step is to:

a. Begin CPR immediately with a compression to breath ratio of 3:1
b. Begin CPR immediately with a compression to breath ratio of 5:1
c. Bag valve mask (BVM) ventilate for 1 minute. If he remains bradycardic, begin chest compressions at that time.
d. Bag valve mask (BVM) ventilate for 5 minutes. If he remains bradycardic, begin chest compressions at that time.

18. You are called to the home of a 4 year old having an asthma attack. Which of the following places a child at high risk of severe asthma exacerbation?

a. Family history of asthma
b. No prior use of controller medications
c. No prior admissions for asthma
d. Previous ICU admission for asthma

19. A 20 month old child does not respond to your presence. She is breathing 12 times per minute and her pulse rate is 60bpm. She has been having difficulty breathing during the past 12 hours. You should:

a. Assist ventilations with a bag valve mask (BVM)
b. Have the parent administer supplemental oxygen using the blow-by technique
c. Place her in a sitting position and open her airway
d. Administer high-fiow oxygen via a non-rebreather mask

20. End-tidal CO_2 monitoring is an important tool to detect changes in ventilation. End-tidal CO_2 monitoring detects apnea or hypoventilation before a patient's pulse oximeter demonstrates hypoxia. End-tidal CO_2 monitoring is useful in which of the following scenarios?

a. In the post-ictal child. Elevated CO_2 is consistent with effective ventilation.
b. In the post-ictal child. Elevated CO_2 or absent tracing detects a hypoventilating child.
c. In the actively seizing child. Elevated CO_2 is consistent with effective ventilation.
d. In the actively seizing child. Absent tracing is consistent with effective ventilation.

BLS providers, please proceed to the reverse side of the answer sheet.
Questions 21-25 are for ALS.

21. During resuscitation of a newborn, you've established adequate ventilation with an endotracheal tube and your colleague has begun chest compressions for a heart rate under 60bpm. Nevertheless, after 60 seconds, the heart rate has not increased. What is the most appropriate next step in management?

a. Administer epinephrine
b. Discontinue chest compressions
c. Start a dopamine infusion
d. Stimulate the newborn

22. You are called to an unplanned home birth for a newborn at 31 weeks gestation. You are having difficulty ventilating despite using a BVM with an appropriately sized mask. What size uncuffed endotracheal tube should be used to intubate this neonate?

a. 2.5mm
b. 3.0mm
c. 4.0mm
d. 4.5mm

23. Which technique is considered to be the gold standard for confirming ET tube placement?

a. Absent sounds on auscultation of the epigastrium
b. Visualizing rise and fall of the chest
c. Clear and equal breath sounds on auscultation of the chest
d. Use of recordable waveform capnography

24. A 10kg infant is in cardiac arrest. The monitor shows ventricular fibrillation. For the first shock, you administer:

a. 5 joules
b. 10 joules
c. 20 joules
d. 150 joules

25. You are the paramedic managing the resuscitation of a pulseless newborn. Currently, chest compressions and bag valve mask ventilations are being performed. You have attempted 2 IVs unsuccessfully. What is the best next step to effectively administer epinephrine in this situation?

a. Give 0.01mg/kg of 1:1,000 (1mg/mL) epinephrine IM
b. Place endotracheal tube and give 0.01mg/kg of 1:1,000 (1mg/mL) epinephrine ET
c. Place IO and give 0.01mg/kg of 1:10,000 (0.1mg/mL) epinephrine IO
d. Attempt new IV and give 0.01mg/kg 0f 1:10,000 (0.1mg/mL) epinephrine IV

ALS providers, please proceed to the reverse side of the answer sheet.
